# Decoding dengue’s neurological assault: insights from single-cell CNS analysis in an immunocompromised mouse model

**DOI:** 10.1186/s12974-025-03383-w

**Published:** 2025-03-04

**Authors:** Minyue Qiu, Lixin Zhao, Xiaojia Li, Yipei Fan, Minchi Liu, Dong Hua, Yunkai Zhu, Yinyin Liang, Yu Zhang, Wen Xiao, Xiaofeng Xu, Jintao Li

**Affiliations:** 1 Department of Biosafety, School of Basic Medicine, Army Medical University, Chongqing, China; 2 Institute of Immunology, Army Medical University, Chongqing, China

**Keywords:** Dengue encephalitis, Single-nucleus RNA sequencing, GABAergic neurons, Immune cell infiltration, CD8^+^ T cells, Neurological impairment

## Abstract

**Background:**

Dengue encephalitis, a severe neurological complication of dengue virus infection, is increasingly recognized for its rising incidence and significant public health burden. Despite its growing prevalence, the underlying mechanisms and effective therapeutic strategies remain poorly understood.

**Methods:**

Cellular atlas of dengue encephalitis was determined by single-nucleus RNA sequencing. Viral load of dengue virus and the level of cytokines expression was detected by RT-qPCR. The target cells of dengue virus were verified by immunofluorescence. The cytotoxic effect of CD8^+^ T cell was determined by flow cytometry, immunofluorescence, in vivo CD8^+^ T cell depletion, adoptive transfer and CCK-8-based cell viability assay. Axonal and synaptic reduction induced by dengue virus infection was demonstrated by RT-qPCR, Western blot, transmission electron microscope and immunofluorescence. Finally, motor and sensory functions of mice were detected by open field test and hot plate test, respectively.

**Results:**

In this study, we utilized single-nucleus RNA sequencing on brain tissues from a dengue-infected murine model to construct a comprehensive cellular atlas of dengue encephalitis. Our findings identify neurons, particularly inhibitory GABAergic subtypes, as the primary targets of dengue virus. Additionally, immune cell infiltration was observed, contributing to significant neurological damage. Comprehensive analyses of cell-cell communication, combined with CD8^+^ T cell depletion and transfer restoration experiments, have elucidated the critical role of CD8^+^ T cells in triggering encephalitis through their interaction with neurons. These cells infiltrate the brain from peripheral circulation, interact with neurons, and induce damage of synapse and axon, accompanied by neurological dysfunction.

**Conclusion:**

We defined cellular atlas of dengue encephalitis in mouse model and identified the primary target neuron of dengue virus. In addition, we demonstrated the significant cytotoxic effect of CD8^+^ T cell, which leads to apoptosis of neuron and neurological dysfunction of mice. Our study provides a molecular and cellular framework for understanding dengue encephalitis through advanced sequencing technologies. The insights gained serve as a foundation for future investigations into its pathogenesis and the development of targeted therapeutic approaches.

**Supplementary Information:**

The online version contains supplementary material available at 10.1186/s12974-025-03383-w.

## Introduction

Dengue virus (DENV), a member of the Flaviviridae family, is a single-stranded RNA virus that significantly impacts global health [1]. It is estimated to cause nearly 100 million symptomatic cases annually, affecting a population of approximately 400 million individuals who are at risk [2, 3]. The primary vectors for DENV transmission are *Aedes albopictus* and *Aedes aegypti* [4, 5]. With escalating global warming, dengue cases are increasing, concurrently enhancing the comprehension of the associated pathogen-induced diseases. In 2009, the World Health Organization (WHO) refined the classification of symptomatic DENV infections, transitioning from the Dengue Fever (DF), Dengue Hemorrhagic Fever (DHF) and Dengue Shock Syndrome (DSS) [6] to: Dengue without warning signs (D), Dengue with warning signs (DWS), and Severe Dengue (SD) [7–10], reflecting an advanced understanding of the disease. DENV-associated neuropathologic features are listed as one of the clinical indicators of SD [11].

Neurological manifestations of DENV, primarily encephalitis and encephalopathy, have been frequently reported, alongside rarer conditions such as brachial neuritis, optic neuritis, Guillain-Barre syndrome, myositis, myoclonus syndrome, and Parkinson-like symptoms [12–15]. Dengue encephalitis, in particular, is noted for its high mortality rate and severe prognosis, thus representing one of the most critical neurological complications of DENV infection [16–19]. It presents with a spectrum of clinical signs such as reduced consciousness, headaches, fever, nausea, vomiting, neurological deficits, and coma, often accompanied by seizures, convulsions, paralysis reminiscent of polio, and Parkinson’s-like motor disturbances [20, 21]. Diagnosis of dengue encephalitis is typically achieved through a multimodal approach, integrating PCR analysis for the detection of viral RNA in serum or cerebrospinal fluid, serological assays for the identification of DENV-specific antibodies, and the utilization of neuroimaging for a comprehensive clinical evaluation [22–25]. The presence of viral antigens in the brain tissue of deceased patients indicates that DENV can invade the Central Nervous System (CNS) and is associated with the manifestation of neuropathologic symptoms [26, 27].

It is well-established that various Flaviviruses can induce neurological symptoms [28, 29]. Although DENV is not classically categorized as a neurophilic virus like Japanese Encephalitis Virus (JEV) or Zika Virus (ZIKV), its significant neurological impact and noteworthy neurotropic characteristics are increasingly recognized [30–33]. However, the mechanisms underlying Flavivirus-induced CNS damage remain obscure, leading to challenges in recognizing disease progression and a lack of effective prevention or treatment strategies [29].

Research on DENV-induced encephalitis remains in its early stages, with significant gaps in understanding the susceptibility of CNS cells to DENV infection. While studies have shown that various cell types—including endothelial cells, microglia, and neurons—can be vulnerable to DENV infection [34–37], in vivo findings suggest that direct endothelial cell infection may be limited. Instead, activated endothelial cells are thought to contribute to pathogenesis indirectly by amplifying inflammatory responses in coordination with infiltrating immune cells [38]. Brain biopsy analyses from fatal dengue cases indicate a diverse range of infected cell types, including neurons, microglia, astrocytes, endothelial cells, Purkinje cells, and choroid plexus cells [39–41]. These observations highlight the complexity and heterogeneity of DENV encephalitis pathogenesis, emphasizing the need for a more comprehensive understanding of CNS cell susceptibility, immune cell interactions, and the mechanisms of tissue damage following DENV infection. Given that clinical brain tissue samples are typically derived from fatal cases, their availability is limited, and systematic confirmation of infection across different time points is lacking. As a result, the precise mechanisms by which DENV infects CNS cells and initiates neuropathologic changes remain to be elucidated. Sensitive animal models are essential for systematically investigating these processes and confirming the sequence of events leading to encephalitis.

In our previous research, we developed an effective DENV encephalitis model via intranasal inoculation [42]. To elucidate the neurological impacts of DENV through the olfactory route in immunocompromised mice, we employed single-nucleus RNA sequencing (snRNA-seq) to analyze DENV-infected mice brain tissues at the single-cell level. Our findings indicate that various inherent CNS cells, predominantly neurons, are susceptible to DENV post-CNS invasion, with evidence of neuronal loss and functional impairment accompanied by extensive infiltration of both innate and adaptive immune cells, including CD8^+^ T cells which are identified as a major cause of neuronal damage. This study provides a crucial experimental foundation for understanding the neuropathologic mechanisms of DENV-induced CNS disorders in murine model and offers a new perspective for understanding DENV-induced neurological disease.

## Methods

### Cells and viruses

Vero cells were maintained in DMEM medium (Gibco, USA) supplemented with 10% fetal bovine serum (FBS) (VivaCell, China) and cultured at 37 °C with 5% CO_2_ for virus cultivation. Neuro2a cells were maintained in MEM medium (Gibco, USA), supplemented with 10% FBS, 1% Glutamax (Vivacell, China), 1% Sodium Pyruvate (Vivacell, China) and 1% NEAA (eLGbio, China), and cultured at 37 °C with 5% CO_2_. DENV-2 (Dengue virus 2 New Guinea C derivative strain, GenBank accession no. FJ390389.1), which is the most likely serotype to cause SD among four DENV serotypes, was obtained from the Center for Disease Control and Prevention of the Southern Military Theatre. Virus was cultivated and titer tested as previously described [42].

### Animals experiment

Since young individuals have a higher proportion of neurological involvement following DENV infection, four to six-week-old immunocompromised mice named A6 (IFN-α/β receptor-deficient mice) were applied in this study. For infection, A6 mice were anesthetized with ether and intranasally (i.n.) inoculated with 30 µL of DENV-2 (3 × 10^5^ PFU) by pipetting into one nare. Mock group mice were treated with fluid supernatant of brain homogenates from healthy suckling mice. Mice used for snRNA-seq, quantitative-polymerase chain reaction (qPCR), RNA-sequencing (RNA-seq) and western blot experiments, were anesthetized with 1.25% avertin (Tribromoethanol) (Nanjing Aibei, China) and dissected to harvest brain tissue for the follow-up experiment. For Nissl stain, immunofluorescence, TUNEL staining and TEM experiments, mice were perfused and fixed with physiological saline (Cisen, China) and 4% paraformaldehyde (PFA) (BioSharp, China) after anesthesia and dissection. For flow cytometry experiment, mice were perfused by HBSS (Solarbio, China). Since no significant sex-based difference in survival rates was observed in DENV intranasally infected A6 mice in the pilot study, we have chosen to use female mice in our study design to ensure experimental consistency and the stability of behavioral research.

### Nucleus isolation

The brain tissues samples were surgically removed and divided along the midsagittal plane. One half of the mouse brain were snap-frozen in liquid nitrogen for intact nucleus isolation. The nuclei were isolated and purified as previously described, with some modifications [43]. Briefly, the frozen tissue was homogenized in NLB buffer which contain 250 mM Sucrose, 10 mM Tris-HCl, 3 mM MgAc2, 0.1% Triton X-100 (SigmaAldrich, USA), 0.1 mM EDTA, 0.2 U/µL RNase Inhibitor (Takara, Japan). Various concentrations of sucrose were used to purify the nuclei. The concentration of nuclei was adjusted to about 1000 nuclei/µL for snRNA-seq.

### Single-nucleus RNA-Seq experiment

The snRNA-seq libraries were generated using the 10X Genomics Chromium Controller Instrument and Chromium Single Cell 3’ V3 Reagent Kits (10X Genomics, Pleasanton, USA) according to the instructions. Briefly, cells nuclei were concentrated to 1000 nuclei/µL than loaded into each channel of the Chromium Controller to generate single-cell Gel Bead-In-Emulsions (GEMs). After the reverse transcription step, which includes cDNA synthesis at 53℃ for 45 min (min), enzyme inactivation at 85℃ for 5 min, and reaction termination at 4℃, GEMs were mechanically broken and the barcoded-cDNA was purified and then amplified under the following conditions: an initial denaturation at 98 °C for 3 min, followed by 25 cycles of 98 °C for 15 s (sec), 63 °C for 20 s and 72 °C for 1 min, then a final extension at 72 °C for 1 min and held at 4 °C. The amplified barcoded cDNA was subjected to fragmentation, A-tailing and ligation with adaptors. Following this, an index PCR amplification was performed. The final libraries were quantified using the Qubit High Sensitivity DNA assay (Thermo Fisher Scientific, USA) and the size distribution of the libraries were determined using a High Sensitivity DNA chip on a Bioanalyzer 2200 (Agilent, USA). All libraries were sequenced by Novaseq6000 (Illumina, USA) platform on a 150 bp paired-end run.

### Single-cell RNA statistical analysis

We applied fastp (https://github.com/OpenGene/fastp) with default parameter filtering the adaptor sequence and removed the low quality reads to achieve the clean data [44]. The filtering process trims segment R1 to 28 bp. Then the feature-barcode matrices were obtained by aligning reads to the mouse genome (mm10 Ensembl: version 100) using CellRanger (version: 6.1.1, https://support.10xgenomics.com/) with an expected feature count of 10,000, as specified in the analysis parameters. We applied the down sample analysis among samples sequenced according to the mapped barcoded reads per cell of each sample and finally achieved the aggregated matrix. Cells contained over 200 expressed genes and mitochondria UMI rate below 10% passed the cell quality filtering and mitochondria genes were removed in the expression table.

Seurat package (version: 4.0.3, https://github.com/satijalab/seurat) was used for cell normalization and regression based on the expression table according to the UMI counts of each sample and percent of mitochondria rate to obtain the scaled data. PCA was constructed based on the scaled data with top 2000 high variable genes and top 10 principals were used for tSNE construction and UMAP construction. Utilizing graph-based cluster method, we acquired the unsupervised cell cluster result based the PCA top 10 principal and we calculated the marker genes by FindAllMarkers function with wilcox rank sum test algorithm under following criteria:1. lnFC Threshold > 0.25; 2. min.pct > 0.1; 3. Resolution > 0.8. To identify the specific cell types, clusters consisting of identical cell types were selected for re-tSNE analysis, graph-based clustering, and marker analysis. R package (version: 3.6.2, https://www.r-project.org) was used for datasets analyzing and visualizing. The “pheatmap” package was employed to generate heatmap, the “ggplot2” package was adopted for its versatility in creating sophisticated plots, such as scatter plots, violin plots, and box plots. The synergistic use of these tools with R package formed the basis of our visualization strategy.

### Cell communication analysis

To enable a systematic analysis of cell–cell communication molecules, we applied the Cellchat tool (version 1.1.3, http://www.cellchat.org/), which establishes the probability of cell-to-cell communication by integrating prior knowledge of the interactions between gene expression and signaling ligands, receptors and their cofactors. Briefly, cell communication is calculated at the ligand-receptor level, then the communication probability at the pathway level is calculated by summarizing the communication probability of all the ligand-receptor interactions associated with each cell pathway, and finally, the converged cell communication network is calculated by calculating the number of links or summarizing the communication probability. The threshold for P-values is set to 0.05.

### SCENIC analysis

To assess transcription factor regulation strength, we applied the Single-cell regulatory network inference and clustering (pySCENIC, version: 0.9.5, https://aertslab.org/#scenic) [45] workflow, using the 20-thousand motifs database for GRNboost2 with AUC_THRESHOLD: 0.05, NES_THRESHOLD: 3, RANK_THRESHOLD: 5000.

### QuSAGE analysis

Quantitative Set Analysis for Gene Expression (QuSAGE) is a novel gene set enrichment analysis tool. It provides a method that fully considers the activity of gene sets by evaluating the combined effect of all gene variations in a pathway. Unlike methods that assess deviation from the null hypothesis with P-value, QuSAGE employs a complete probability density function (PDF) to quantify the activity of gene sets. To characterize the relative activation of a given gene set such as pathway activation, we performed QuSAGE (version: 2.16.1, https://bioconductor.org/packages/release/bioc/html/qusage.html) analysis by aligning with database (Ensembl100 KEGG96) [46].

### Differential gene expression analysis

To identify differentially expressed genes among samples, the function FindMarkers with wilcox rank sum test algorithm was used under following criteria: (1) lnFC > 0.25; (2) pvalue < 0.05; (3) min.pct > 0.1.

### Viral genome identification

The feature-barcode matrices were obtained by aligning reads to the DENV genome using CellRanger v6.1.1. Cells with viral reads less than 2 are considered to be false positives caused by the contamination of viral genomes during the nucleus isolation process. To verify the accuracy of the analytical results, we employed the Viral-Track software (https://github.com/PierreBSC/Viral-Track) for the detection of viral genomes. This sophisticated computational pipeline is specifically designed for in-depth analysis of viral genomes from single-cell RNA sequencing data [47]. The analysis was conducted following the procedures published on the software’s website. Briefly, it includes: installation of the R package, creation of the index and annotation file, pre-processing of the data, and detection of viruses.

### Pseudo-time analysis

We applied the Single-Cell Trajectories analysis utilizing Monocle2 (version: 2.22.0, http://cole-trapnell-lab.github.io/monocle-release) [48] for pseudo-time analysis. In this analysis, we employed the DDR-Tree as our reduction method with a DDRTree-lambda set to 5. Before Monocle analysis, we select marker genes of the Seurat clustering result and raw expression counts of the cell passed filtering. Based on the pseudo-time analysis, branch expression analysis modeling (BEAM) analysis was applied for branch fate determined gene analysis.

### GO and pathway analysis

Gene ontology (GO) analysis was performed to elucidate the biological implications of marker genes and differentially expressed genes [49]. We downloaded the GO annotations from NCBI (http://www.ncbi.nlm.nih.gov/), UniProt (http://www.uniprot.org/) and the GO (http://www.geneontology.org/). Fisher’s exact test was applied to identify the significant GO categories and FDR was used to correct the p-values. Pathway analysis was conducted to identify significant pathways of marker genes and differentially expressed genes according to KEGG database. We used Fisher’s exact test to select the significant pathway [50], with the threshold of significance defined by P-value and FDR.

### qPCR

Total RNA was extracted from mouse tissues and cells using TRIzol reagent (Tiangen Biotech, Beijing, China). The reverse transcription of total RNA into cDNA was accomplished using the PrimeScript™ RT Reagent Kit (TaKaRa, Tokyo, Japan). Subsequently, qPCR was initiated by combinations of 5µL of TB Green Premix Ex Taq II (TaKaRa, Tokyo, Japan), 4µL of cDNA and 1µL of specific primer. The reaction process was carried out in the LightCycler^®^ 96 system (Roche, Basel, Switzerland). The viral RNA copies per mL was calculated according to quantification cycle (Cq) values and a standard curve established by DENV-2 plasmid with known concentrations. Primers sequences were listed in Supplementary Table 1.

### Nissl stain

Brain tissue was collected and fixed in 4% PFA for 24 h. After gradient ethanol dehydration, xylene transparency, and paraffin embedding, tissue was cut into 5 μm thick sections. It was dewaxed and rehydrated to perform the Nissl staining according to the kit instruction (Solarbio, China). The M8 Digital Scanning Microscopic Imaging System (ZEISS, Germany) was applied to capture images.

### Fluorescence in situ hybridization

For the DENV genomic RNA detection in situ, DENV specific probe were designed according to DENV NS5 genome (probe sequence: 5’ FITC-GCTCCACATTTGGGCGTAGGACTTC). Experiment was performed using an RNA Fluorescence In Situ Hybridization (FISH) Reagent Kit (for Paraffin Sections) (GenePharma, Shanghai, China) according to the manufacturer’s manual. Tissue sections (3 µM thick) were heated at 60 ℃ for 30 min to melt the paraffin. Then sections were immersed in xylene for deparaffinization and rehydrated in an ethanol series. Tissue sections were rinsed by PBS for twice before boiling pretreatment in a water bath for 20 min. After treatment with proteinase K for 30 min at 37 ℃, tissue sections were dehydrated in an ethanol series and membrane permeabilizated by 0.1% Triton X-100 (Sigma-Aldrich, USA) for 10 min. Then, slides were denatured at 78 ℃ for 8 min before detected by DENV specific probe and stained with DAPI (Sigma-Aldrich, USA). Images were obtained with LSM880 laser confocal microscope (ZEISS, Germany).

### Immunofluorescence

After anesthetization, perfusion and fixation, the tissue was harvested and fixed in 4% PFA for 24 h. Subsequently, the fixed tissue was dehydrated with 30% sucrose solution (Beyotime, China) and embedded in optimal cutting temperature compound (OCT) (ThermoFisher, USA) and cut into 5 μm thick sections by cryostat microtome (ThermoFisher CryoStar NX50 Cryostat, USA). After stasis at room temperature for 10 min, sections were washed by PBS (Servicebio, China) for 15 min. Then a blocking solution (5% goat serum and 0.3% Triton X-100 in PBS) (Beyotime, China) was applied to blocking sections at room temperature for 1 h. Next, the sections were incubated with primary antibodies for overnight at 4 °C. After 3 times washing, the sections were incubated with the secondary antibodies (See Supplementary Table 2 for all antibodies applied in this study). Finally, the sections were stained with 4,6-diamidino-2-phenylindole (DAPI) for 7 min. Fluorescent sections were visualized by LSM880 laser confocal microscope (ZEISS, Germany). Statistical analysis of random field cell counts was applied by Fiji v2.9.0 software.

### TUNEL

Terminal deoxynucleotidyl transferase (TdT) mediated dUTP-bitin nick end labeling (TUNEL) staining kit (Roche, Switzerland) was used to detect cell apoptosis. Brain tissue sections were permeablilized in 0.1% Triton X-100 (Sigma Aldrich, USA) for 5 min. Then sections were incubated with TUNEL reaction solution in a dark box at 37 ℃ for 1 h. After DAPI (Sigma Aldrich, USA) staining of cell nuclei, the sections were sealed with anti-fluorescence quenching agent (Beyotime, China) and observed by LSM880 laser confocal microscope (ZEISS, Germany).

### RNA-seq

TRIzol reagent was used for tissue nucleic acid extraction. RNA concentration and integrity were then tested. Qualified samples were applied for library construction. After two rounds of binding mRNA to capture beads, the mRNA was immediately broken into fragments for constructing a library. Detailed operations included the synthesis of the complementary DNA (cDNA), second strand of cDNA, purification, end repair, adenine deoxyribonucleotide (A) addition to the 3’ terminal end, ligation of joints, fragment selection, PCR amplification, purification of PCR products, and quality assessment using the Qsep-400 system. The Illumina NovaSeq 6000 platform (Illumina, San Diego) were used for sequencing of the constructed library.

### Flow cytometry

Collected brain tissues were washed with precooled RPMI 1640 medium (Gibco, USA), and gently ground in 70 μm sieve (Falcon, USA). The ground homogenate was centrifuged at 4 ℃ for 10 min at a speed of 2000 r/min. Cells were resuspended in RPMI 1640 medium, and 100% Percoll solution (GE Healthcare, UK) was added in a 7:3 ratio of cell suspension to 100% Percoll solution. Then, the 70% Percoll solution was slowly and gently added to form a clear division. The double-layered liquid was centrifuged at 2000 r/min, 22 ℃ for 30 min, with acceleration speed as 1 and deceleration speed as 0. The cell layer was then carefully aspirated and the cells were washed with HBSS solution containing 2% FBS for twice. Peripheral blood was lysed with 1X red blood cell lysis buffer (Beyotime, China) for 5–6 min at room temperature. Following centrifugation at 1500 rpm for 5 min, the supernatant was removed. Cells were resuspended in 1 mL PBS (Servicebio, China). After transferring to a new 1.5 mL tube and re-centrifuging under the same conditions, the cells were resuspended and counted using an automated cell counter, yielding a mononuclear cell suspension for further experimental use. The isolated cells were then resuspended in the Fc receptor blocker TruStain FcXTM (BioLegend, USA) and incubated on ice for 10 min. Flow cytometry antibodies were added and incubated with cells on ice for 30 min. BD FACSCanto II flow cytometer (BD, USA) were used for detection. Data were analyzed by FlowJo10.8.1 software.

### CCK-8-based cell viability assay

Neuro-2a cells (RRID: CVCL_0470) were plated in 96 well plate with a density of 5 × 10^3^ cells per well. After the neuro-2a cells adhered to the wall, the Fas blocking group were treated with Fas antibody (Santa, USA) 1 h before viral inoculation. For viral inoculation, the culture medium was removed and treated with DENV (MOI = 10) and incubated at 37℃ for 2 h. The viral solution was then discarded and medium was added in each well. For Fas ligand (FasL) protein treated group, cells were treated with Animal Free Fas Ligand Protein (MedChemExpress, USA) at 24 hpi. Twenty-four hours later, 10 µL of CCK-8 (Beyotime, China) stock solution was added and incubated at 37℃ for 2 h. The optical density (OD) value at 450 nm was measured by BioTek Synergy H1 (BioTek, USA). Cell viability was calculated according to the following formula:

$$\begin{aligned}\text{Cell}\:\text{viability}\:\left(\%\right)&= [ (\text{sample}\:\text{OD}\:\text{value}\:-\:\text{blank}\:\text{well}\:\text{OD}\:\text{value})\\&/(\text{control}\:\text{well}\:\text{OD}\:\text{value}\:-\:\text{blank}\:\text{well}\:\text{OD}\:\text{value})]\\&\:\times\:\:100\%\end{aligned}$$

### In vivo CD8^+^ T cell depletion

A6 mice were randomly divided into two groups: the CD8b.2 antibody-depleted group and the isotype control group. Mice were intraperitoneal (i.p.) injected with 200 µg of anti-mouse CD8b.2 monoclonal antibody (Leinco, USA) for the experimental group and an equivalent dose of isotype control antibody (Selleck, USA) for the control group, administered bi-daily for two rounds. Twenty-four hours post the final depletion, mice were i.n. infected with DENV. Subsequently, peripheral blood was subsequently sampled via the tail vein bleeding method at 3, 5, and 7 days post-infection (dpi) for flow cytometry to verify the efficacy of CD8^+^ T cell depletion. At 7dpi, mice brain tissues were harvested for further experiments.

### Adoptive transfer

At 7dpi, spleens from DENV-infected and mock group A6 mice were harvested and processed into single-cell suspensions by 70 μm strainer. After 10 min of red blood cell lysis at room temperature, the splenocytes were washed, counted and resuspended in sorting buffer at a concentration of 1 × 10^8^ cells/ml. CD8^+^ T cells negative sorting was performed following the manufacturer’s instructions for the Mouse CD8^+^ T-cell Isolation kit (Biosharp, China), and the purity of the sorted cells was confirmed by flow cytometry. Following a 4-day depletion period, CD8-depleted mice and isotype controls were infused with specified CD8^+^ T cell subsets or non-CD8^+^ T cells from DENV-infected or mock group mice (1 × 10^6^ cells/mouse). One day post-infusion, the mice were infected intranasally with DENV, and tissue samples were collected at 7dpi.

### Western blot

Dissected tissues were homogenized in lysis buffer (Beyotime, China) to extract protein. The protein concentration was then quantified using the Bicinchoninic acid (BCA) protein assay (Beyotime, China) kit. The quantified protein was denatured at 100 ℃ for 5 min and loaded into an SDS polyacrylamide gel (EpiZyme, China) for electrophoresis. After that, protein was transferred onto a PVDF membrane (Beyotime, China) from the selected area of the PAGE gel for 2 h. Next, the PVDF membrane was blocked with 1% BSA (Solarbio, China) for 1 h and then incubated with primary antibodies at 4 ℃ for at least 16 h. After thoroughly washing, the PVDF membrane was incubated with secondary antibodies for 1 h. Finally, the PVDF membrane was treated with ECL substrate (Solarbio, China) and the luminescent signal was detected.

### Transmission electron microscope (TEM)

After perfusion, brain tissues were collected and fixed by 3% glutaraldehyde (shyuanye, China) overnight. The tissues were then fixed by 1% osmic acid for 2 h. Next, the tissues were dehydrated using acetone and embedded in resin. After ultrathin section (70 nm) by diamond blade, the sections were stained using 2% uranyl acetate and 0.4% lead citrate. Images were obtained using a transmission electron microscope (FEI TECNAI G2 12, Thermo Fisher Scientific, US).

### Immunocytochemistry (ICC)

After 48 h of virus infection, the medium of neuro-2a cells were discarded. Then, cells were washed with PBS and fixed with 4% PFA. One hour later, cells were penetrated with 0.1% Triton X-100 (Sigma Aldrich, USA) for 10 min after thoroughly washing. Then, cells were blocked with 10% goat serum (Beyotime, China) for 1 h. Next, primary antibodies were added and incubated at 4 ℃. The next day, secondary antibodies were added and incubated at room temperature for 1 h after thoroughly washing with PBS. Cells were visualized by LSM880 laser confocal microscope (ZEISS, Germany).

### Open field test (OFT)

To assess the activity capacity of mice at 7dpi, a 500 × 500 × 350 mm OF were set as 16 equally squares to record the animal trace. After placing the mouse in the OF facing the corner of the box, recording of the animal activity was started immediately and monitored for 10 min. Then, the mice were put back into the breeding cages. A Video analysis system for joint opening (ZSDichuang, China) was applied in this study.

### Hot plate test (HPT)

To evaluate the sensory function changes of mice after DENV infection at 5dpi, 7dpi, 9dpi and 14dpi. Mice were placed on a 55 ℃ hot plate (BME-480, China) and confined with a transparent acrylic cylinder. The latency of the reaction (i.e. the duration of licking hind paw or jumping) were measured immediately. To protect the animals, the longest monitored period is 60 s. After the measurement was completed, the mice were returned into the breeding cages. Hot plate was wiped with 20% alcohol and dried before the next round of test.

### Statistical analysis

Data were analyzed by GraphPad Prism software (version: 9.4.0) and Fiji software (version: 2.9.0). Data were shown as mean ± SEM. T-test was utilized to compare the means of two groups of data. Statistical significance was set at *p* < 0.05.

## Result

### Cell cluster identification in DENV-infected mouse brain via snRNA-Seq

**Fig. 1:**
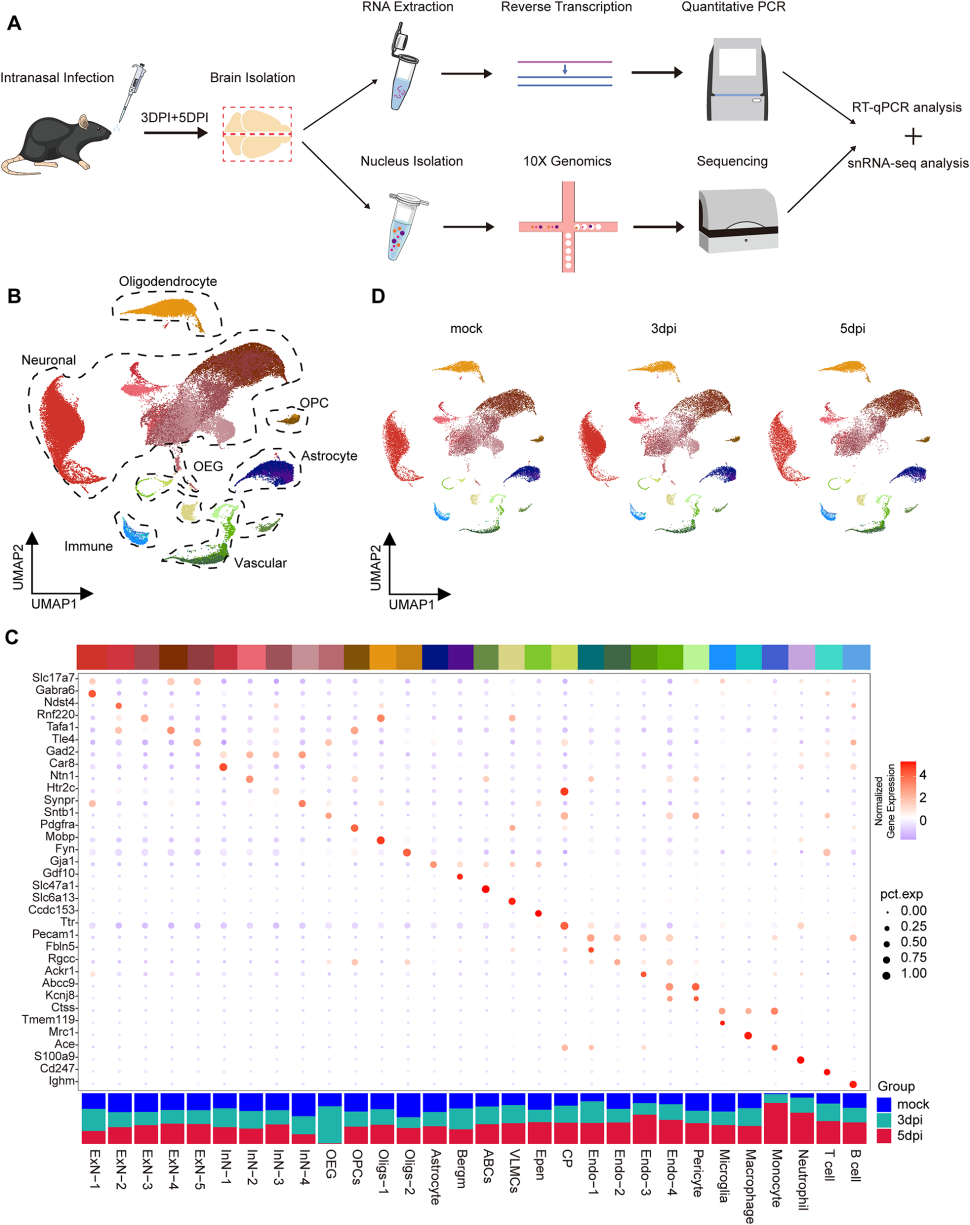
snRNA-seq revealed cell atlas of DENV invaded immunocompromised mouse central nervous system. (**A**) Experimental scheme. (**B**) Uniform manifold approximation (UMAP) visualization of cells from all samples. (**C**) Cell types determined by snRNA-seq clustering analysis. Including the example marker genes expression of different cell types (the intermediate bubble chart) and fraction of cells per cluster (the barplots at the bottom). (**D**) UMAP plots of mock, 3dpi and 5dpi group (*n* = 3)

DENV promotes immune evasion and viral proliferation in humans by inhibiting the interferon signaling pathway. However, wild-type mice are naturally resistant to DENV infection, limiting their utility for studying DENV pathogenesis. Interferon receptor-deficient mice, which lack functional interferon signaling, thus serve as a valuable model for investigating DENV pathogenesis [51]. To investigate the CNS landscape following DENV invasion via the respiratory tract, we utilized type I interferon receptor knockout mice. These mice were intranasally infected with DENV serotype 2, as detailed in our methods [42]. Post-inoculation, the animals were euthanized at 3 or 5 days, and their brain tissues were dissected for analysis. One half of the brain underwent qPCR to quantify viral loads and to measure the expression of acute inflammatory markers, such as IL-1β, TNFα, and IL-6, at the aforementioned time points (Fig. 1A). Significant detection of viral RNA and elevated levels of these inflammatory markers were observed in infected brain tissues (Supplementary Fig. 1A and 1B), confirming successful intranasal inoculation and subsequent infection.

The other half brain tissue samples were subjected to snRNA-seq (Fig. 1A). To discern dynamic transcriptomic changes in various brain cell types post-DENV infection, we used mock group as a baseline. Nuclei from all samples were extracted and sequenced using 10X Genomics technology. After quality control (Supplementary Fig. 1C), a total of 81,610 cells were included in our analysis: 28,302 cells from mock group, 27,256 cells from mice at 3dpi, and 26,052 cells from mice at 5dpi. On average, this corresponded to 8,000–10,000 cells per sample.

Utilizing Uniform Manifold Approximation and Projection (UMAP) analysis through the Seurat package in R, we identified 29 major cell clusters (Fig. 1B). Cell types were categorized based on known marker genes, encompassing major neuronal, glial, vascular, and immune cells (Fig. 1C, Supplementary Fig. 1D). The neuronal clusters included excitatory (ExN) (glutamatergic) and inhibitory (InN) (GABAergic) neuronal subtypes, defined by specific gene expression patterns. Glial clusters were characterized as olfactory ensheathing glial (OEG), oligodendrocyte precursor cells (OPCs), oligodendrocytes (Oligs), astrocytes (Astro), and Bergmann glial cells (Bergm). Non-neural clusters encompassed arachnoid barrier cells (ABCs), vascular leptomeningeal cells (VLMCs), ependymal cells (Epen), choroid plexus cells (CP), endothelial cells (Endo), pericytes (Peri), microglia (Micro), macrophages (Macro), monocytes (Mono), neutrophils (Neut), and lymphocytes (T and B cells) (Fig. 1C).

A noticeable reduction in cell count, particularly in neuronal and OEG populations, was observed following DENV infection (Fig. 1C and D). Conversely, an increasing trend was noted in vascular and immune cell counts (Fig. 1C and D). However, other glial cell numbers remained relatively stable, despite some fluctuations (Fig. 1C and D). Our analysis aligns with previous large-scale studies of brain cell types, wherein neurons constituted the majority (approximately 70%), followed by oligodendrocytes (8.3%) and astrocytes (7.6%) [52, 53]. This concordance suggested that our dataset accurately reflects the cellular composition of the brain tissues under study.

### Pronounced neuronal alterations following DENV intrusion

**Fig. 2:**
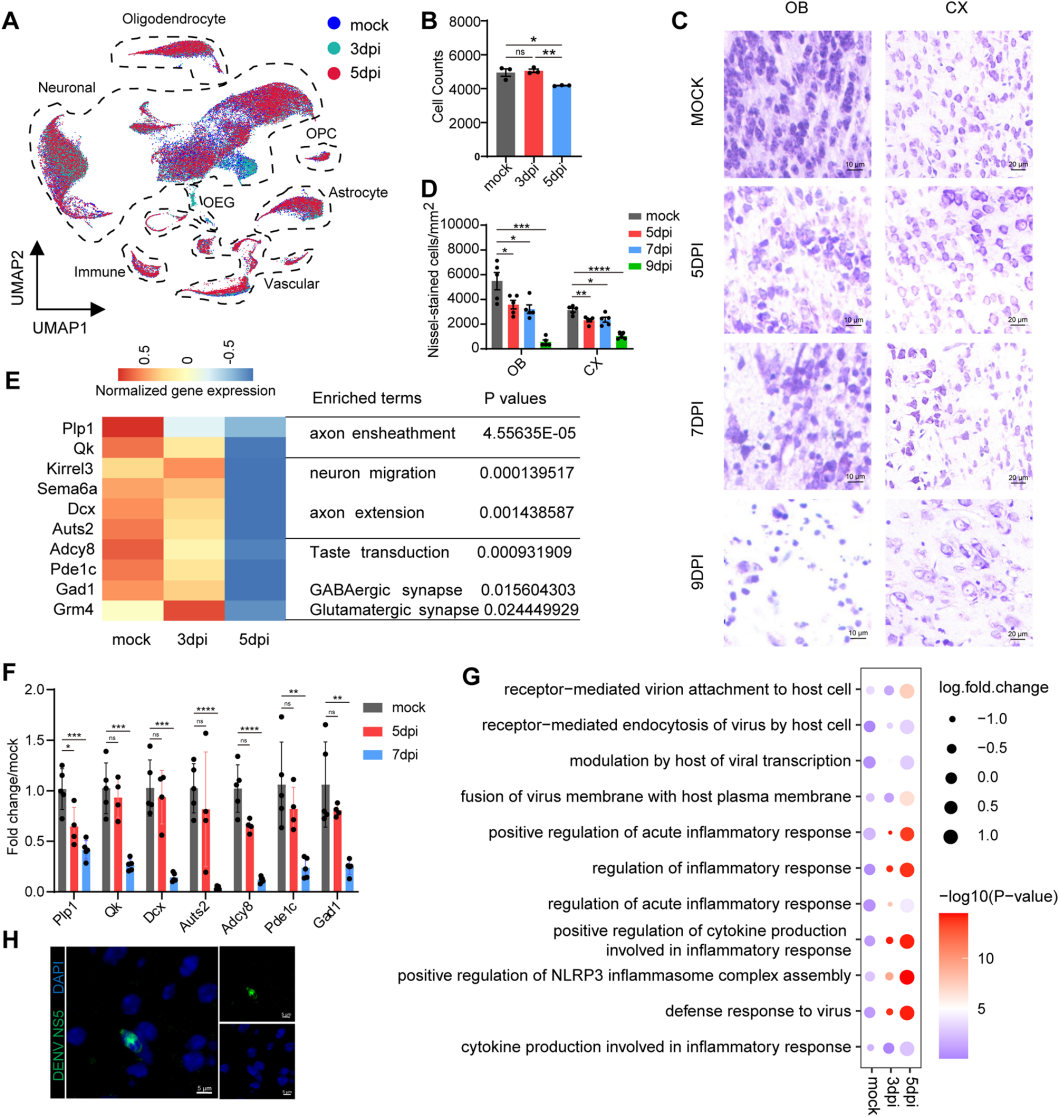
Changes in neuronal count and state of mouse across DENV infection. (**A**) Cells density UMAP plots of three groups. (**B**) Density of neurons across the three groups identified by snRNA-seq analysis. (**C**) Representative Nissl staining images of DENV infected mice brain tissue at different time points. Scale bar: 10 μm (OB), 20 μm (CX). (**D**) Statistical graphic of Nissl staining in (**C**) (*n* = 5). (**E**) Heatmap of distinct genes enriched in neurons. Distinct genes (left) related to Mock group are highlighted with enriched gene ontology (GO) biological process terms (right top) and Kyoto Encyclopedia of Genes and Genomes (KEGG) terms (right bottom). Red represents a higher expression level, and blue represents a lower expression level. (**F**) Quantification of mRNA expression level of genes in (**E**) in mice OB (*n* = 4–5). (**G**) Bubble plot of relative enrichment of virus attachment and entry relative gene sets (left top) and immune response relative gene sets (left bottom) between different groups as computed via QuSAGE analysis. Each group was evaluated separately. (**H**) Representative FISH images showing DENV NS5 labeled with fluorescent probes and colocalized with DAPI in mouse OB at 5dpi (green: DENV NS5, blue: DAPI). Scale bar: 5 μm. *indicates p value < 0.05, **indicates p value < 0.01, ***indicates p value < 0.001, ****indicates p value < 0.0001. Data are presented as mean ± SEM

Previous studies have established the susceptibility of neurons to DENV infection both in vivo and in vitro [37]. In this study, we focused on the impact of DENV on neuronal populations. Our analysis based on snRNA-seq data revealed a significant decline in overall neuronal cell numbers as the infection progressed (Fig. 2A). While the neuron count at 3dpi did not show a significant difference compared to the mock group, a notable decrease of 16% was observed at 5dpi (Fig. 2B). Nissl staining further corroborated these findings, indicating substantial neuronal loss in various brain regions such as the cerebral cortex (CX) and olfactory bulb (OB) starting from 5dpi (Fig. 2C and D). This neuronal loss was observed at a later stage in the hippocampal regions, including the Cornu Ammonis (CA) and Dentate Gyrus (DG) areas (Supplementary Fig. 2A and B).

To delve deeper into the molecular changes within neurons post-infection, we analyzed gene expression levels between the 5dpi and mock groups. This analysis identified 64 differentially expressed genes (DEGs) in the 5dpi group. GO and Kyoto Encyclopedia of Genes and Genomes (KEGG) pathway analyses revealed a downregulation in genes associated with synaptic function, axonal structure, neuronal migration, and taste perception (Fig. 2E). These findings were further supported by qPCR (Fig. 2F). Additionally, we employed QuSAGE analysis to assess the antiviral activity within neurons, discovering the activation of multiple antiviral pathways and acute inflammatory responses at 5dpi. Notably, the stages of the viral infection process, from attachment to membrane fusion, were significantly activated (Fig. 2G).

Then we attempt to quantify viral load at the single-cell level by aligning viral genomes with all samples based on unique molecular identifiers (UMIs). Although it is well known that single-stranded RNA viruses primarily replicate in cell cytoplasm, analysis via Cellranger revealed the identification of 2,425 viral genome fragments within the annotated cells from our final group annotation. Of these, a predominant 95.38% were derived from neuronal clusters, followed by lower proportions from oligodendrocytes (1.24%) and astrocytes (1.11%) (Supplementary Fig. 2C). Parallel analyzed results obtained by the Viral-Track software [47], a method for globally scanning unmapped scRNA-seq data to detect viral RNA, are basically consistent with those of cellranger (Supplementary Fig. 2D). Intriguingly, RNA sequences coding for all DENV proteins were detected in the infected samples, with non-structural protein 5 (NS5) being the most prevalent (Supplementary Fig. 2E). To validate the bioinformatics results, DENV NS5 RNA probes were designed and applied in mouse brain tissue sections. Result showed that there were obvious co-localization of viral RNA and nucleus (Fig. 2H). This result indicated that nucleus translocation of DENV genome might commonly occurred in highly infected cells.

### Elevated susceptibility of inhibitory neurons to DENV

**Fig. 3:**
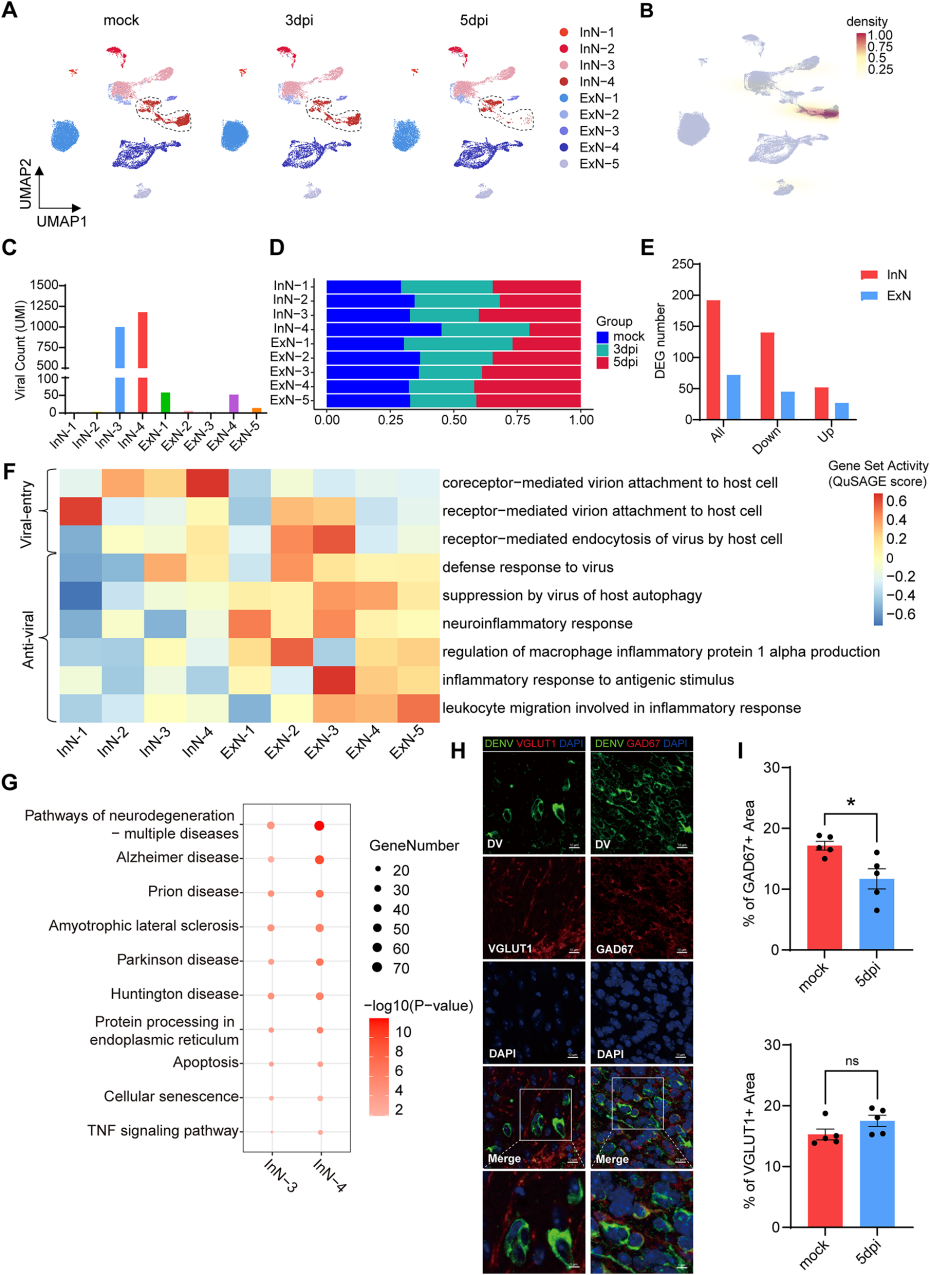
Subcluster specific susceptibility of DENV in neuron. (**A**) Neuronal individual UMAP plots with dotted line circles the significantly loss cell subpopulations. (**B**) Viral UMAP plots, shown as the density of viral UMI count overlaid on total cell population across all samples (gray). (**C**) Statistical graphic of viral count in (**B**). (**D**) The proportion of cells in each neuronal cluster of mock, 3dpi and 5dpi group in (**A**). (**E**) Quantification of the number of DE genes for ExN and InN. (**F**) Heat map of the enrichment of virus attachment and entry relative gene sets (right top) and inflammatory responses relative gene sets (right bottom) between different neuronal subclusters. Red represents activation, and blue represents inhibition. (**G**) KEGG pathway enrichment in VGNAs distinct to other cells within their respective clusters. (**H**) Representative immunofluorescence images of the co-localization of inhibitory or excitement neuron with viruses in mouse OB at 5dpi. (green: DENV, red: excitement neuron labeled by VGLUT1 or inhibitory neuron marker by GAD67, blue: DAPI). Scale bar: 10 μm. (**I**) Statistical graphic of inhibitory or excitement neuron proportion in (**H**) (*n* = 5). *indicates p value < 0.05. Data are presented as mean ± SEM

In exploring the specificity of DENV for different neuronal subtypes, our analysis focused on neuronal subclusters (Fig. 3A, Supplementary Fig. 3A and 3B). We observed that viral genome nucleus accumulation predominantly occurred in inhibitory neuronal clusters InN-3 and InN-4, with viral genome counts of 999 (43.19%) and 1177 (50.89%), respectively (Fig. 3B and C). Notably, the cell counts in the InN-4 cluster showed a significant decrease post-infection, whereas no substantial change was observed in the overall InN-3 cluster (Fig. 3D).

To assess the differential impact of DENV on target cells, we compared gene expression between the infected and mock groups. The analysis revealed a higher number of DEGs in inhibitory neurons compared to excitatory neurons, across all categories of DEGs, whether upregulated or downregulated (Fig. 3E). Further investigation into the disparity in DENV susceptibility between excitatory and inhibitory neurons focused on virus-host interaction and immune response pathways. While receptors mediating viral attachment and endocytosis were partially activated in both neuron types, a bias toward inhibitory neurons was noted in coreceptor-mediated viral attachment (Fig. 3F). Interestingly, despite the low detection of viral genomes in the nuclei of excitatory neurons, the activation level of the pathway of defense response to virus is higher in excitatory neuronal subpopulations compared to inhibitory neurons. In addition, autophagy processes in excitatory neurons were suppressed, coupled with a consistently activated inflammatory response (Fig. 3F). These findings suggest that an inactive inflammatory response post-early virus exposure could contribute to the increased susceptibility of inhibitory neurons to DENV.

To avoid the inaccuracy results caused by viral sequence contamination during the nucleus isolation process, cells with a detected nucleus viral load at least 2 UMIs were classified as viral genome nucleus accumulated cells (VGNAs). Given the highest detection of viral genomes in InN-3 and InN-4 clusters, the transcriptomic profiling of VGNAs was conducted on these two groups. Following the DEGs analysis between VGNAs and other cells within their respective clusters, KEGG pathway analysis revealed significant enrichment of pathways related to neurodegenerative diseases, apoptosis, and the TNF signaling pathway in the VGNAs (Fig. 3G).

To further corroborate the specific neurotropism of DENV for certain neuronal types, infected mice were euthanized and dissected at 5dpi for immunofluorescence analysis. The results demonstrated significant DENV antigen (labeled by protein E of DENV) co-localizing with inhibitory neurons (labeled by GAD67) at 5dpi, whereas minimal co-localization was observed with excitatory neurons (labeled by vGlut1) (Fig. 3H). Quantification across five random fields revealed a marked decrease in inhibitory neuron count following infection in OB (Fig. 3I). These findings underscored that, although all neuronal subpopulations exhibit notable changes post-DENV invasion, inhibitory neurons predominantly serve as the major target for viral intrusion.

### Activation of multiple apoptotic mechanisms in neurons post-DENV intrusion

**Fig. 4:**
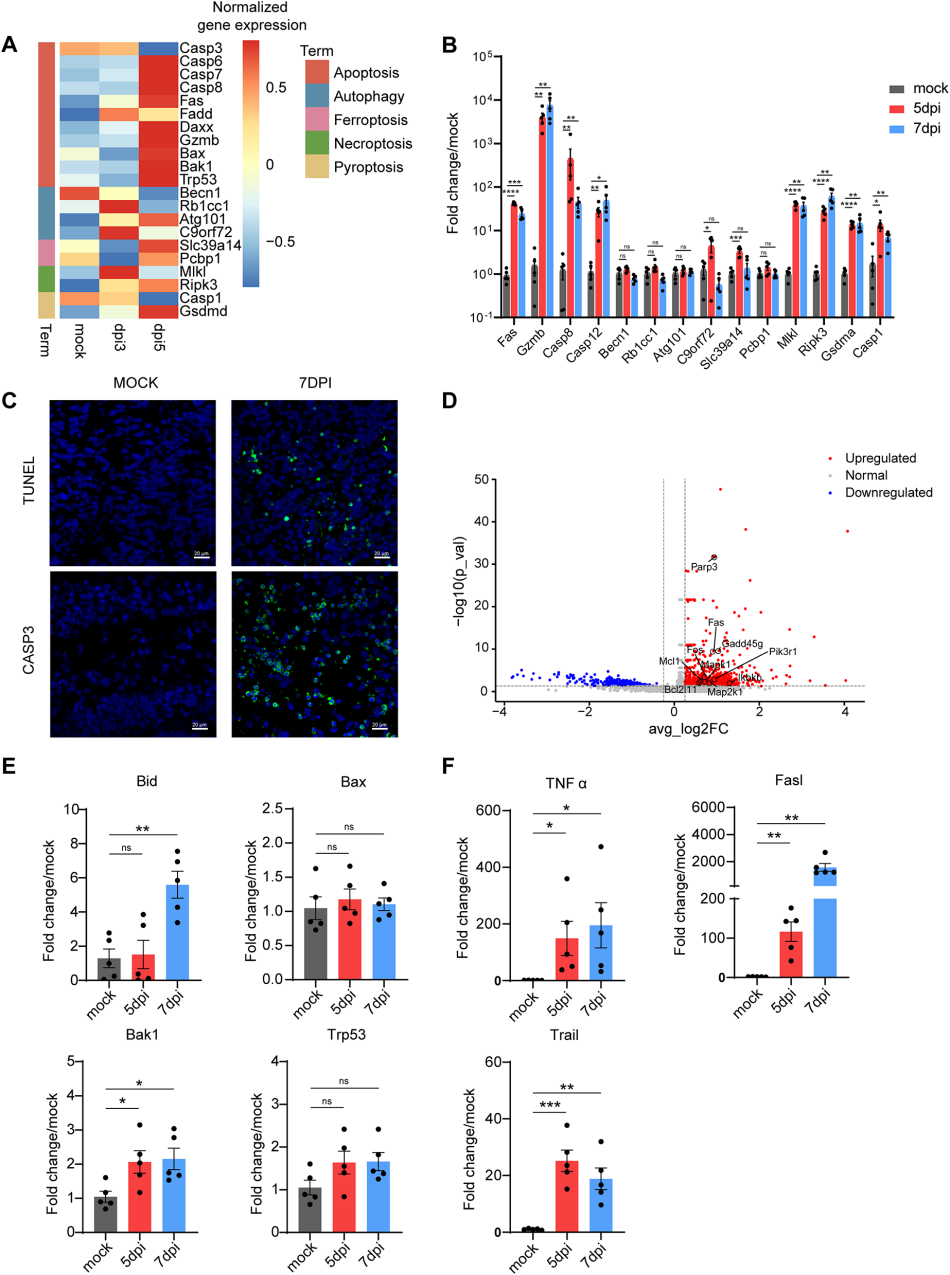
The exogenous apoptotic signaling pathway represented by Fas-FasL showed significant activation in DENV infected brain tissue. (**A**) Expression of genes related to apoptosis, autophagy, ferroptosis, necroptosis and pyroptosis in neuron of mock, 3dpi and 5dpi group. Red represents a higher expression level, and blue represents a lower expression level. (**B**) Quantification of mRNA expression level of genes in (**A**) in mice OB (*n* = 5). (**C**) Representative immunofluorescence images of TUNEL and cleavaged caspase 3 in mock or DENV infected mice OB at 7dpi. Scale bar: 20 μm. (**D**) Expression of enriched genes in apoptosis pathways between VGNAs and other cells in InN-4 cluster of mock, 3dpi and 5dpi group. (**E**) Quantification of mRNA expression level of endogenous apoptosis regulators in mice OB (*n* = 5). (**F**) Quantification of mRNA expression level of exogenous apoptosis inducing molecules genes in mice OB (*n* = 5). *indicates p value < 0.05, **indicates p value < 0.01, ***indicates p value < 0.001, ****indicates p value < 0.0001. Data are presented as mean ± SEM

Consistent with prior studies indicating that DENV infection induces cell apoptosis through various pathways [54, 55], our study observed a notable decrease in neuronal cell counts in mouse brain tissue following DENV infection (Fig. 2B and C). Considering the spectrum of cell death mechanisms, including apoptosis, pyroptosis, necrosis, autophagy, and ferroptosis, we analyzed the expression levels of the selected genes of these pathways in KEGG database using our snRNA-seq data. Post-infection, a widespread upregulation of apoptosis pathway selected genes was evident in neurons (Fig. 4A). Further validation through qPCR revealed expression of the selected genes in the apoptotic pathway were generally higher than those in other death pathways after DENV infection (Fig. 4B). Since OB is the first CNS invasion site of DENV through olfactory route, we used RNA-seq to investigate the transcriptome changes of mice OB after DENV invasion. Apoptosis pathway was also evident in RNA-seq analyses of infected mouse OB (Supplementary Fig. 4A). Immunofluorescence staining for TUNEL and cleaved caspase-3 corroborated these findings, confirming the apoptotic state of brain tissues post-DENV infection (Fig. 4C).

To elucidate the interactions among apoptosis pathway genes, we utilized the online tool String to analyze gene enrichment from our RNA-seq data. This analysis simulated protein interactions, identifying Fas, caspase-8, and c-FLIP (Cflar) as central mediators in multiple apoptosis-inducing factors, including TNF, FasL, granzyme, and stress-induced pathways (Supplementary Fig. 4B and 4 C). To further explore the differences in apoptosis pathways between VGNAs and other cells within their respective clusters, we analyzed the enriched genes in apoptosis pathways between VGNAs and other cells in the InN-4 cluster and found that expression of extrinsic apoptosis pathway initiation genes Fas, tumor necrosis factor receptor superfamily member 6, was significantly upregulated in DENV positive cells. Other significantly upregulated apoptosis-related genes include the intrinsic apoptosis pathway-related gene Bcl2l11, the Mapk1 which can function in both extrinsic and intrinsic apoptosis pathways, the DNA damage response-related genes Parp3 and Gadd45g, the anti-apoptotic genes Mcl1, Pik3r1, and Ikbkb, as well as the apoptosis-related signal-regulating genes Fos and Map2k1 which do not directly participate in the apoptotic pathways (Fig. 4D). Consistently, the results of qPCR showed that intrinsic apoptosis regulators, including the pro-apoptotic Bcl-2 family genes Bid, Bax, and Bak1, along with the pivotal transcription factor Trp53, demonstrated negligible differences from the mock group at 5dpi. Until 7dpi, there was a modest upregulation of these genes (Fig. 4E). For the exogenous apoptosis inducing molecules, the expression of FasL was upregulated more significant than Trail and TNF-α, as infection increasing (Fig. 4F). These results collectively suggested that multiple apoptotic mechanisms are initiated in neurons following DENV infection, with a notable emphasis on the exogenous pathway mediated through death receptors.

### Interactions between infiltrated immune cells and neurons predominantly mediated by Fas-FasL axis

**Fig. 5:**
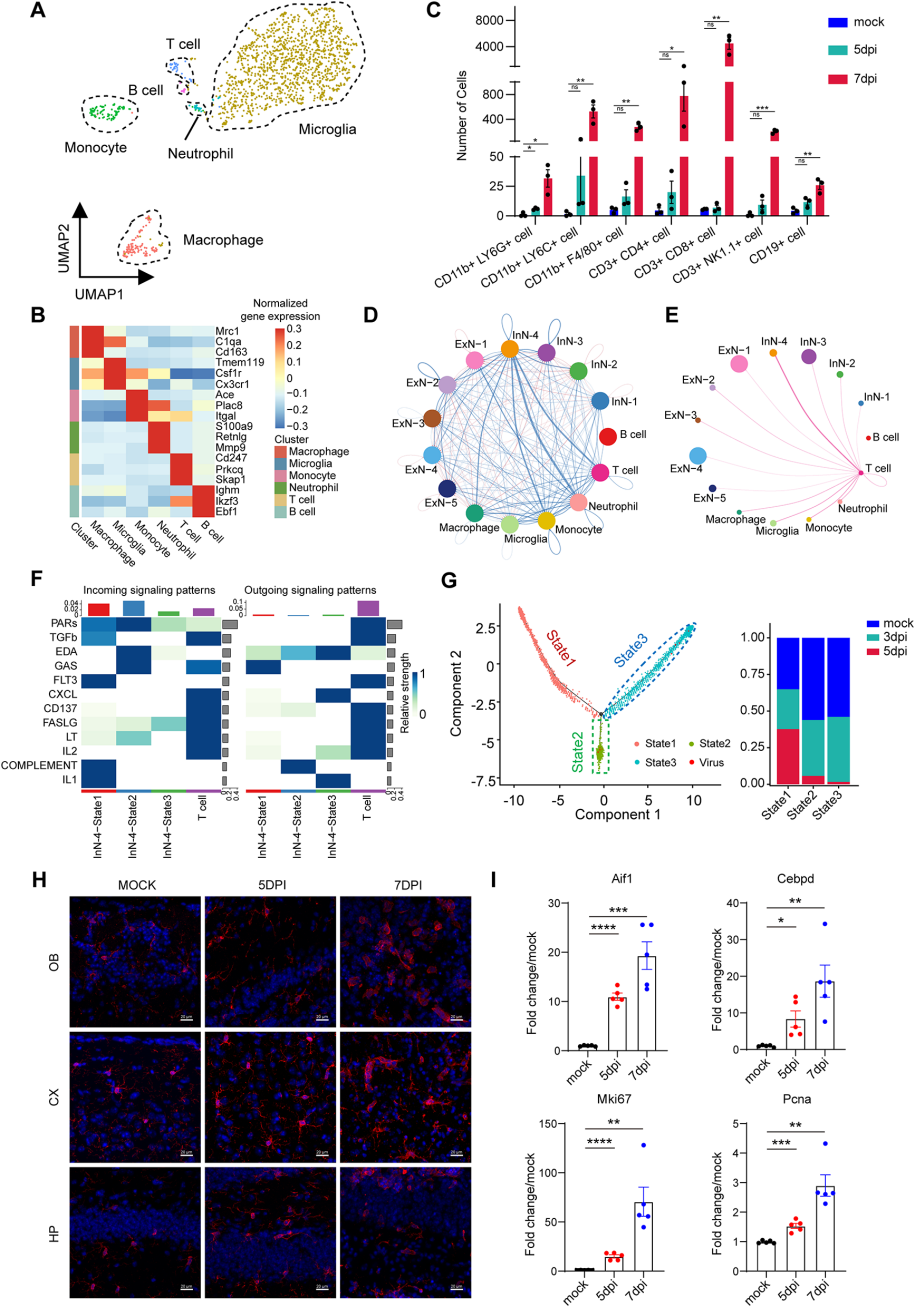
Characterization of immune cells in mock and DENV infected mouse brain. (**A**) UMAP visualization of immune cells from all samples. (**B**) Heat map of immune cell clusters with unique signature genes. Red represents a higher expression level, and blue represents a lower expression level. (**C**) Flow cytometry of the immune cells number and cell types in DENV infected mouse brain (*n* = 3). (**D**) Circle plot of differential interaction strength between immune cells and neuronal subclusters, depicting interaction potential in 5dpi group. (**E**) Circle plot of interaction weight between T cells and neuronal subclusters in 5dpi group. (**F**) Intercellular communication of immune response and cell injury relative pathway between T cell and 3 states of InN-4 cluster cells. The heatmap showed the relative importance of each cell cluster based on the computed network centrality measures of outgoing/incoming signaling network. The column chart displayed the relative contribution of each cluster or pathway. (**G**) Single-cell trajectories of the InN-4 cluster by Monocle analysis (left). The states circled by dashed lines represent states enriched with VGNAs. The proportion of InN-4 cells in each state (right). (**H**) Representative immunofluorescence images of microglia (marked by Iba-1) in mock-infected or DENV infected mice brain. (**I**) Quantification of mRNA expression level of microglial activation and proliferation-associated genes in the mouse OB at 5dpi. Scale bar: 20 μm. *indicates p value < 0.05, **indicates p value < 0.01, ***indicates p value < 0.001. Data are presented as mean ± SEM

FasL (FASLG, CD95L) is a member of the tumor necrosis factor (TNF) family and serves as a ligand for TNFRSF6/FAS. Expressed in a variety of cell types including T cells, natural killer (NK) cells, monocytes, neutrophils, breast epithelial cells, and vascular endothelial cells [56]. FasL plays a pivotal role in immune responses. To ascertain the source of FasL in mouse brain tissue post-DENV infection, we examined immune cell populations using snRNA-seq. Our analysis identified six major immune cell clusters: Macrophages (Mrc1^+^C1qa^+^CD163^+^) [57], Microglia (Tmem119^+^Csf1r^+^Cx3cr1^+^) [58–60], Monocytes (Ace^+^Plac8^+^Itgal^+^) [61], Neutrophils (S100a9^+^Retnlg^+^Mmp9^+^) [62, 63], T cells (Cd247^+^ Prkcq^+^Skap1^+^) [64–66], and B cells (Ighm^+^ Ikzf3^+^Ebf1^+^) [67, 68] (Fig. 5A and B).

To complement the snRNA-seq data and address its limitations in fully characterizing immune cells, we conducted flow cytometry on brain tissue from infected mice [69]. Using CD45 as a marker, we identified neutrophils (CD11b^+^Ly6G^+^), macrophages (CD11b^+^F4/80^+^), monocytes (CD11b^+^Ly6C^+^), CD4^+^ T cells (CD3^+^CD4^+^), B cells (CD19^+^), NKT cells (CD3^+^NK1.1^+^), and CD8^+^ T cells (CD3^+^CD8^+^) (Supplementary Fig. 5A). The immune cell types detected at 5dpi were largely consistent between flow cytometry and snRNA-seq results (Fig. 5C, Supplementary Fig. 5B). However, flow cytometry identified higher cell counts, suggesting an undersampling of immune cells in snRNA-seq, possibly due to the small size and fragility of their nuclei [70]. Notably, a significant change in immune cell composition was observed at 7dpi, with a marked increase in CD8^+^ T cells, CD4^+^ T cells, neutrophils, monocytes, and NKT cells (Fig. 5C).

To explore the activation of apoptosis signaling pathways, we analyzed the interactions between neurons and immune cells using CellChat. This analysis revealed extensive and complex interactions between inhibitory neurons and various immune cells at 5dpi (Fig. 5D). T cells, known to generate cytotoxicity via FasL and granzyme release, showed strong interactions with the InN-4 cluster (Fig. 5E). Their inflammatory effects in regulating immune responses were significantly more pronounced than those of other immune cell types (Supplementary Fig. 5C). A heatmap illustrating the relative importance of cell clusters in activating injury-associated signaling pathways indicated that T cells were predominant in outgoing signaling, while incoming signaling pathways were activated in different states of the InN-4 cluster. This suggests that T cells interact with neurons through various signaling pathways (Fig. 5F). Among these, FASLG exhibited strong interaction with state 3 InN-4 neurons, which showed significant cell loss (Fig. 5F and G). The BEAM plot illustrates the pseudotime trajectory of the InN-4 cluster, highlighting the unique gene expression profiles across different cell populations (Fig. 5G). Specifically, within the state2 and state3 which characterized by VGNA cell aggregation and a notable reduction in neuronal count, the expression of various apoptosis-associated genes within the corresponding modules is markedly elevated compared to that observed in state 1 (Supplementary Fig. 5D).

Although snRNA-seq has been used to identify subpopulations of microglia in this study, it is not suitable for detecting the activation status of microglia due to technical limitations [71]. Therefore, to investigate the status of microglia, which were reported to exhibit immune regulatory and cytotoxic functions as resident immune cells in the CNS [72], we employed immunofluorescence in the DENV infected mice. The results revealed that microglia activation was evident by 7dpi, characterized by enlargement of cell bodies, shortening of protrusions, and a shift to circular or rod-shaped cell morphology (Fig. 5H). The qPCR verification further confirmed these observations, demonstrating a modest upregulation of genes associated with microglial activation (Aif1, Cebpd) and proliferation (Pcna, Mki67) in the mouse OB at 5dpi. The activation intensity was substantially increased by 7dpi (Fig. 5I). This temporal change suggested that microglia may play a more prominent role in the later stages of DENV-induced CNS pathology.

### CD8^+^ T cells mediated neuropathology in DENV infection

**Fig. 6:**
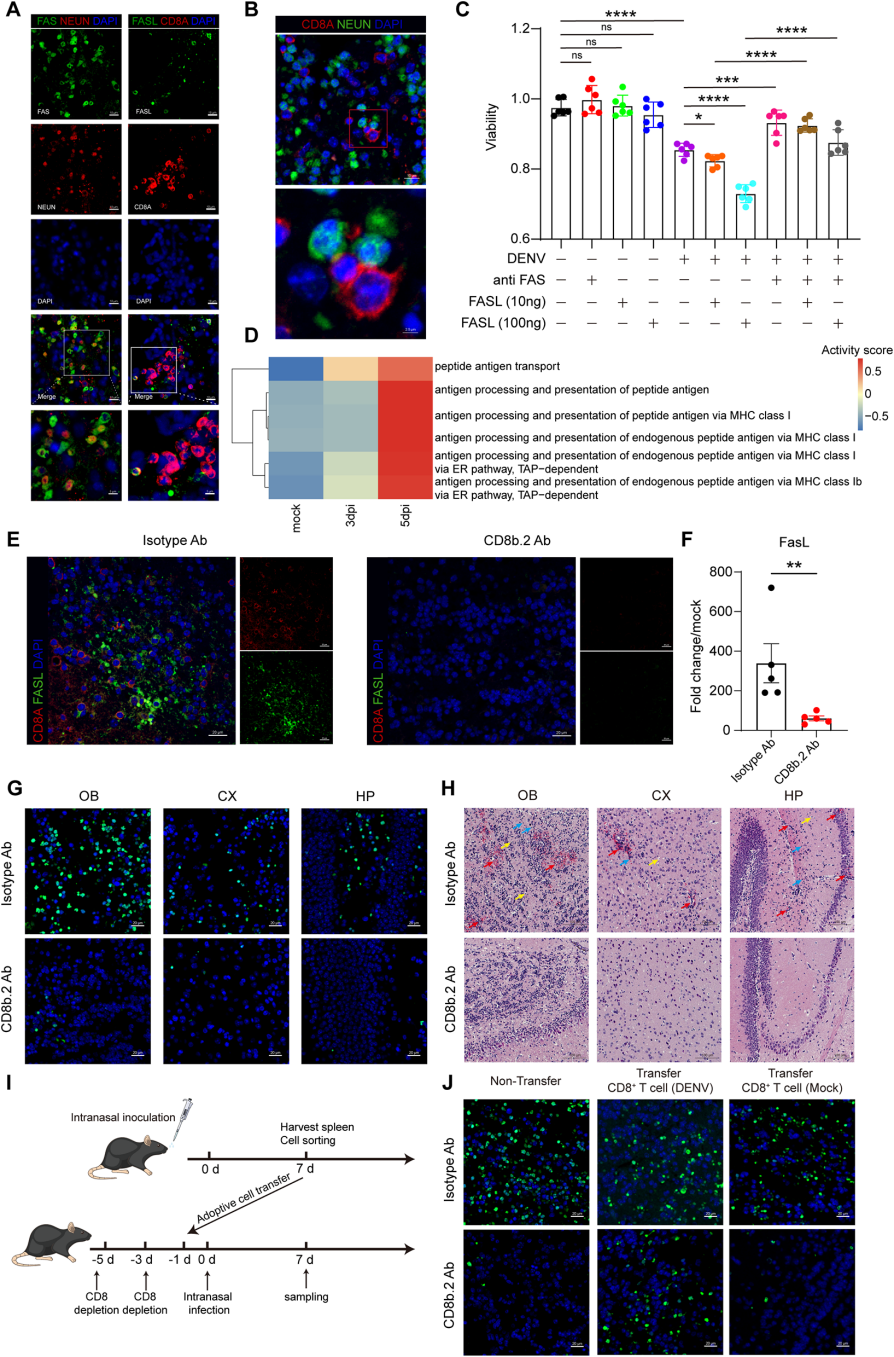
Infiltrated CD8^+^ T cells cause the neuronal damage after DENV infection. (**A**) Representative immunofluorescence images of Fas^+^NeuN^+^ cells and FasL^+^CD8a^+^ cells in DENV invaded mice OB. Scale bar: 10 μm. Magnified view scale bar: 5 μm. (**B**) Representative immunofluorescence images of the co-localization of CD8^+^ T cells (labeled by CD8a) and neuron (labeled by NeuN). Scale bar: 10 μm. Magnified view scale bar: 2.5 μm. (**C**) Cell viability of neuro-2a which treated with DENV, Fas antibody, and FasL protein according to the experimental design (*n* = 6). (**D**) Heat map of the enrichment of antigen processing and presentation relative gene sets at different time point. Red represents activation, and blue represents inhibition. (**E**) Representative immunofluorescence images of the CD8a and FasL in isotype control antibody treated mice OB (left) or anti-CD8b.2 antibody depleted mice OB (right) at 7dpi. Scale bar: 20 μm. (**F**) Quantification of mRNA expression level of FasL in mice OB at 7dpi (*n* = 5). (**G**) Representative TUNEL staining images of isotype control antibody treated mice OB (up) or anti-CD8b.2 antibody depleted mice OB (down) at 7dpi. Scale bar: 20 μm. (**H**) Representative HE staining images of isotype control antibody treated mice brain (up) or anti-CD8b.2 antibody depleted mice brain (down) at 7dpi. Scale bar: 100 μm. The red arrow represents hemorrhage, the blue arrow represents inflammatory cell infiltration, and the yellow arrow represents neuronal death. (**I**) Experimental scheme of adoptive transfer. (**J**) Representative TUNEL staining images of OB in adoptive cell-transferred mice at 7dpi. Scale bar: 20 μm. *indicates p value < 0.05, ***indicates p value < 0.001, ****indicates p value < 0.0001. Data are presented as mean ± SEM

To further investigate the role of Fas-FasL interactions in CNS pathology post-DENV infection, immunofluorescence staining was performed on brain tissue of infected mice. We observed a significant increase in FasL, predominantly co-localized with CD8a at 7dpi (Fig. 6A). Correspondingly, the expression of Fas in brain tissues notably increased, mainly co-localized with the neuronal marker NeuN (Fig. 6A). Additionally, caspase3 and caspase8, apoptosis associated factors widely accumulated in neurons (Supplementary Fig. 6A). To validate the interactive relationship between CD8^+^ T cells and neurons, we examined the spatial distribution of CD8a^+^ cells and NeuN^+^ cells on mouse brain tissue sections at 7dpi. Our findings revealed a close proximity between CD8^+^ T cells and neurons, with observable contacts [73]. These contact zones suggested intercellular interaction between these two cell types (Fig. 6B).

To elucidate the neurotoxic effect of Fas-FasL interactions, neuro-2a cells, a mouse neuroblastoma cell line, were infected with DENV at a multiplicity of infection (MOI) of 10. Post-infection, RNA-seq and qPCR analyses confirmed the activation of apoptotic signaling pathways, similar to our in vivo findings (Supplementary Fig. 6B). Fas-FasL axis-related genes were significantly upregulated following DENV inoculation (Supplementary Fig. 6C). Additionally, neuro-2a cell viability, detected by CCK-8 assay, markedly decreased at 48 h post-infection (hpi) with DENV, further declining upon addition of FasL protein for 24 h. Pre-treatment with Fas antibodies prior to DENV infection increased cell viability at 24 hpi, comparable similar to cells treated with FasL protein alone. This indicated that the engagement of the Fas-FasL pathway following DENV infection is associated with neuron death (Fig. 6C). Analysis of snRNA-seq data showed that the expression levels of MHC I molecule-encoding genes, as well as the activity of neuronal MHC class I-associated pathways, were generally upregulated following DENV infection (Fig. 6D and Supplementary Fig. 6D).

To explore the role of CD8^+^ T cells in DENV-mediated neurological damage, we depleted CD8^+^ T cells in A6 mice using an anti-CD8b.2 antibody, followed by i.n. infection with DENV (Supplementary Fig. 7A). Depletion significantly reduced CD8^+^ T cells counts in peripheral blood (Supplementary Fig. 7B) and eliminated CD8a^+^ cells in brain tissue by 7dpi (Fig. 6E), accompanied by downregulation of the expression levels of FasL protein and mRNA in the OB (Fig. 6E and F). In terms of neuropathological changes, the depleted mice showed better vitality after DENV infection (Supplementary Video), and apoptotic signals in various regions of the brain tissue were significantly reduced (Fig. 6G). HE staining indicated that hemorrhage, inflammatory cell infiltration, and neuronal death in various regions of the mouse brain tissue had essentially disappeared after antibody depletion (Fig. 6H). We then conducted adoptive transfer experiments to assess the direct contribution of CD8^+^ T cells to neurological outcomes following DENV challenge (Fig. 6I). The results showed that neuronal damages were restored in CD8-depleted A6 mice after adoptive transfer of CD8^+^ T cells (with a purity of 94.8%, Supplementary Fig. 7C) extracted from the spleens of infected mice during the acute phase of viral infection (Fig. 6J). However, when depleted mice were infused with mock-treated CD8^+^ T cells (with a purity of 93.4%, see Supplementary Fig. 7C), no significant infiltration of CD8^+^ T cells or apoptosis was observed in the mouse brain tissue (Fig. 6J and Supplementary Fig. 7D). These results indicated that DENV-driven CD8^+^ T cells play an important role in acute phase neuropathology associated with DENV infection.

### DENV infection led to axonal and synaptic damage accompanied by neurological symptoms of mice

**Fig. 7:**
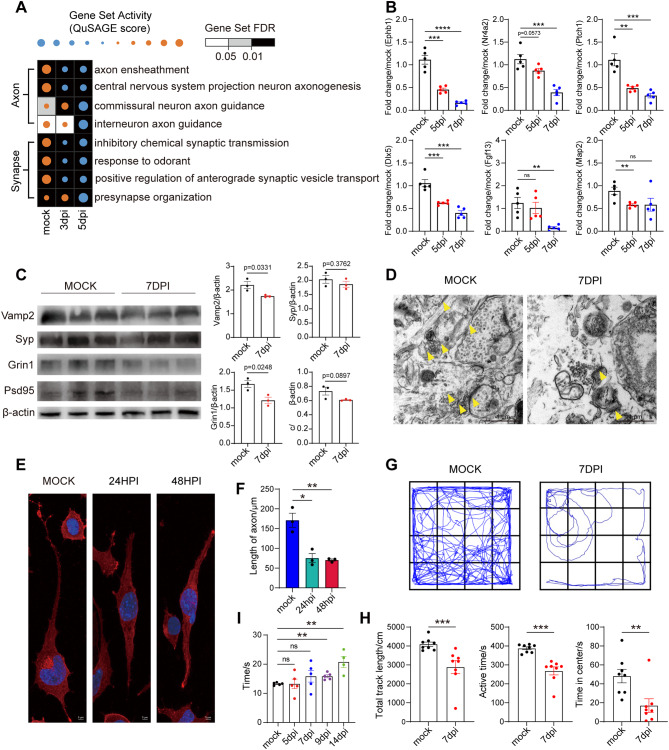
DENV infection causes reduction of axon and synapse, accompanied by animal behavioral disorders. (**A**) Axon (top) and synapse (bottom) relative gene sets activity of neurons at different time point. Red represents activation, and blue represents inhibition. (**B**) Quantification of mRNA expression level of genes in (**A**) in mice OB (*n* = 5). (**C**) Synapse key structural protein levels in DENV infected or mock mouse OB showed by western blot analysis at 7dpi (left). Statistical graphic of corresponding protein level (right) (*n* = 3). (**D**) Representative TEM images of DENV infected or mock mouse OB at 7dpi. Yellow arrows mark synapses, characterized by the presence of synaptic vesicles and the synaptic cleft (the space between presynaptic and postsynaptic membranes). Scale bar: 1 μm. (**E**) Representative immunofluorescence images of neuro-2a with Snap25 labeled synapse and axon. (**F**) Statistical graphic of axonal length in (**E**) (*n* = 3). Scale bar: 5 μm. (**G**) Representative mice trace images of the mock mice and DENV infected mice at 7dpi (*n* = 8). (**H**) Statistical graphic of total track length, motion time and time in center. (**I**) Statistical graphic of hot response time of mice (*n* = 5). *indicates p value < 0.05, **indicates p value < 0.01, ***indicates p value < 0.001. Data are presented as mean ± SEM

Aligned with reports that Fas-FasL pathway activation contributes to axon and synapse damage [74], we analyzed axon and synapse-related pathways in DENV-infected neurons. Pathways related to axoneme assembly, axonogenesis, synapse organization, and synapse assembly were significantly downregulated as the infection progressed (Fig. 7A). Correspondingly, the mRNA expression levels of these pathways-related genes significantly decreased over the course of infection, which were verified by qPCR (Figs. 2F and 7B). Subsequently, we performed a protein-level assessment of the critical synaptic structure proteins, including presynaptic membrane-associated protein Vamp2, Syp, and postsynaptic membrane-associated protein Grin1, Psd95.Western blot analysis showed a downward trend both in presynaptic and postsynaptic membrane-associated protein expression by 7dpi (Fig. 7C). Electron microscopy of neuron synapses revealed a significant reduction in synaptic numbers and notable substructural damage post-infection in mice (Fig. 7D). In DENV-infected Neuro2a cells, qPCR results showed significant downregulation of synaptic-related genes (Supplementary Fig. 8A), and immunocytochemistry showed that synaptosomal-associated protein 25 (Snap25) accumulation were significantly reduced at 24hpi (Fig. 7E). Since Snap25 plays a central role in the activity of the presynaptic membrane, it indirectly reflects the structural and functional characteristics of the axon [75]. By measuring the length of the axons in neuro-2a cells labeled with Snap-25, we found that, there was an average axon length reduction of 55.94% and 58.83% compared to controls at 24hpi and 48hpi, respectively (Fig. 7E and F).

To assess the impact of viral infection on mouse behavior, we conducted OFT and Hot plate test (HPT) in the DENV infected A6 mice. The OFT measures general activity and mental state by recording movement time, residency, and trajectory [76]. The HPT evaluates sensory function, pertinent to assessing the nervous system’s impact [77]. OFT results indicated apathy and reduced mobility in the DENV-infected A6 mice by 7dpi, with average motion time being 69% of the uninfected group. Entry time into the central grid of the OFT arena was significantly reduced in the infected group (Fig. 7G, 7H and Supplementary Fig. 8B). Furthermore, sensory disturbances were evident at 9dpi, with a 57% increase in hot plate response time compared to the mock group at 14dpi (Fig. 7I). These results suggested that DENV CNS invasion affects both motor and sensory functions, aligning with clinical symptoms observed in dengue encephalitis [25, 78, 79].

## Discussion

Exploring the single-cell transcriptomic alterations in the CNS following DENV infection is crucial for advancing our understanding of dengue encephalitis pathogenesis and the broader spectrum of flavivirus-associated neurological disorders. In this study, we present a comprehensive cell atlas of DENV encephalitis in a model of non-brain-invasive infection. This atlas serves as a valuable resource for in-depth exploration of the mechanisms underlying flavivirus-induced neural damage and disease progression from a systemic perspective.

Our findings demonstrate that neurons are the primary target cells of DENV following CNS invasion in immunocompromised mice. The interaction between viral aggression and immune responses leads to neuronal activation, triggering death pathways, downregulating essential functional genes, and manifesting neurodegenerative-like characteristics. Detailed analysis further revealed a specific vulnerability of inhibitory neurons to DENV and subsequent cell loss. Given the critical role of GABA, a key inhibitory neurotransmitter in the CNS, in maintaining neural system stability and balance, the disruption of GABAergic neuron function may be linked to neurological diseases such as epilepsy, a prominent symptom in dengue encephalitis [80]. This suggests that DENV-induced damage to GABAergic neurons and subsequent GABA dysregulation could be a primary factor in the clinical manifestations of dengue encephalitis.

Comparisons between VGNAs and other cells within their respective clusters revealed significant differences, yet both exhibited clear signs of cellular damage. This indicates that neuronal loss in dengue encephalitis results from a combination of direct viral effects and host immune responses. Additionally, a small number of viral genomes have also accumulated in other CNS intrinsic cellular nuclei, such as oligodendrocytes, microglia, and endothelial cells, consistent with the clinical cases observation [81].

To investigate DENV’s destructive impact on intrinsic brain cells, we employed snRNA-seq, effectively capturing transcriptomic data from neurons with large cell bodies. However, the limitations of snRNA-seq in detecting intracellular transcriptomes were apparent, as reflected in the low detected expression levels of intracellular factors. To address this issue, we combined snRNA-seq with flow cytometry for a more comprehensive analysis of immune cells infiltration in brain tissues affected by DENV. Although DENV, as an RNA virus, is believed to replicate in the cytoplasm [82, 83], viral genome fragments, particularly NS5, were prominently detected in the snRNA-seq dataset. Literature review indicates that certain viral RNAs can enter the nucleus and regulate viral replication and host cell antiviral responses relying on a variety of nuclear envelope interactions [84–86]. Specifically, DENV NS5 has been shown to translocate to the nucleus and interact with Daxx, thereby influencing host cell apoptosis [87]. NS5, as DENV’s largest protein, exhibits multiple enzymatic activities, including N-terminal methyltransferase (MTase) for RNA capping and C-terminal RNA-dependent RNA-polymerase (RdRp) for viral RNA synthesis. NS5’s interaction with host factors, known to suppress immune responses and facilitate viral infection, is well-documented [88, 89]. However, the specific role of NS5 in the neurological disease process, particularly its widespread nucleus translocation in neurons, remains to be elucidated and warrants further investigation.

Contrasting with earlier findings, our study reveals that microglia activation was not significant in the early stages (5dpi) of DENV infection in the CNS following olfactory pathway inoculation in mice, which indicated the primary cytotoxic effects caused by infiltrating CD8^+^ T cells. We hypothesize that this discrepancy may be due to the different initial viral concentrations in the brain tissue between non-invasive and invasive DENV infections. In olfactory inoculation, the early-stage viral load and replication rates are lower compared to direct intracranial injections, potentially delaying microglial activation as the virus primarily transmits between neurons. However, by 7dpi, we observed marked activation of microglia. This suggests that in non-invasive dengue encephalitis models, CNS intrinsic immune cells like microglia may play a less dominant role in immunity regulation and viral clearance during the initial stages, possibly as a mechanism for the CNS to avoid excessive immune responses. At later infection stages, the extensive activation of microglia, along with immune cell infiltration, likely contributes to a cytokine storm in the CNS, culminating in the manifestation of neurological disease symptoms.

While previous studies have identified multiple pathways through which DENV infection promotes apoptosis, including the p53 pathway, NFκB-TNF-α pathway, Sphingosine-kinase-2 pathway, ROS-induced DNA damage, and ER stress [90–93], these investigations predominantly utilized animal or human cell lines, such as human hepatocarcinoma cells (Huh7, HepG2), dendritic cells, and endothelial cells. Comprehensive studies specifically focusing on neuronal apoptosis in neural cell lines or experimental animals remain scarce. Consequently, the mechanisms underlying DENV-induced neuronal apoptosis, particularly in the context of CNS invasion, are still not fully understood. Notably, FasL was newly revealed as SD biomarkers in WHO-defined SD patients’ serum [94]. CD8^+^ T cells, crucial for eliminating pathogen-infected cells, originate from hematopoietic stem cells in the bone marrow and mature within the thymus. Upon encountering antigens presented by MHC class I molecules, these cells activate and diversify into distinct effector and memory phenotypes, exerting a substantial influence on the modulation of immune responses [95]. In addition, CD8^+^ T cells have been shown to mediate direct killing of virus-infected cells through the Fas-FasL axis in previous studies [96]. Multiple studies have highlighted the complex and diverse roles of CD8^+^ T cells in various diseases [97, 98]. In this study, neuronal apoptosis was demonstrated to be predominantly mediated by CD8^+^ T cells. The precise phenotypes of CD8^+^ T cells that are critical to DENV-induced neurological injuries remain to be further explored. Interestingly, when we transferred non-CD8^+^ T cells, the apoptotic signal in the brain tissue of depleted mice also increased compared to depleted mice (Supplementary Fig. 7E), suggesting that CD8^+^ T cells are not the only factor mediating neuronal damage. A significant increase in the number of other immune cells infiltrating in the later stages of the disease led to an unbalanced immune system that also contributes to disease severity.

Neuronal signaling fundamentally depends on axonal and synaptic structures. Previous research has highlighted the importance of Fas-FasL pathway activation in axonal and synaptic damage [74]. Our findings indicate a significant reduction in neuronal axons and synapses following DENV infection, characterized by decreased axonal and synaptic generation and assembly. This degradation parallels tissue damage in the mouse brain, leading to neurological symptoms such as sensory impairment and reduced autonomous activity, aligning with clinical manifestations of dengue encephalitis [25, 99]. Notably, the etiology of behavioral disorders in mice is likely multifactorial, extending beyond the evident synaptic and axonal damage. Other critical mechanisms, such as myelin loss, astrocyte activation, and neurotransmitter imbalances, may also significantly contribute to these behavioral changes. For example, astrocytes are key responders to viral infections, capable of secreting pro-inflammatory cytokines that drive CNS inflammation. Additionally, imbalances in excitatory and inhibitory neurotransmitters, such as elevated glutamate and reduced GABA levels, can induce neuronal hyperexcitability and further exacerbate behavioral deficits. These multifactorial interactions underscore the complexity of CNS pathology following infection and highlight the need for further investigation into the interplay between these mechanisms [86, 100, 101].

In conclusion, our study provides a detailed CNS cellular atlas in a DENV-sensitive infection immunocompromised mice model, identifying target cells and characterizing the cellular response at single-cell resolution. We highlight the significant role of CD8^+^ T cells in DENV-mediated neurological damages. However, the potential limitations of using an IFNAR^−/−^ mouse model are noteworthy. IFNα/β interact with CD8^+^ T cells through various mechanisms, including facilitating their activation and proliferation, modulating the expression of cellular adhesion molecules and chemokines, influencing the migration and homing of CD8^+^ T cells, and shaping the development of long-term immunological memory. The absence of IFN signaling in our model may have altered the immune response, potentially affecting the susceptibility and behavior of CD8^+^ T cells during DENV infection. This underscores the necessity for future research utilizing clinical samples to better understand the complexities of human dengue encephalitis and to bridge the translational gap between findings in murine models and their human implications.

## Electronic supplementary material

Below is the link to the electronic supplementary material.

Supplementary Material 1

Supplementary Material 2

Supplementary Material 3

## Data Availability

Raw snRNA-seq data generated in this study are available at NCBI Gene Expression omnibus database (GEO) under accession code GSE252515. RNA-seq data reported in this study are available at NCBI Sequence Read Archive database (SRA) under accession code PRJNA1058718 (mice olfactory bulb) and PRJNA1058860 (neuro-2a cell).

## Abbreviations

DENV: Dengue virus
WHO: World Health Organization
DF: Dengue Fever
DHF: Dengue Hemorrhagic Fever
DSS: Dengue Shock Syndrome
D: Dengue without warning signs
DWS: Dengue with warning signs
SD: Severe Dengue
CNS: Central Nervous System
JEV: Japanese Encephalitis Virus
ZIKV: Zika Virus
snRNA-seq: Single-nucleus RNA sequencing
FBS: Fetal bovine serum
i.n.: Intranasally
qPCR: Quantitative-polymerase chain reaction
RNA-seq: RNA-sequencing
PFA: Paraformaldehyde
GEMs: Gel Bead-In-Emulsions
min: Minutes
sec: Seconds
QuSAGE: Quantitative Set Analysis for Gene Expression
PDF: Probability density function
BEAM: Branch expression analysis modeling
GO: Gene ontology
Cq: Quantification cycle
FISH: Fluorescence In Situ Hybridization
OCT: Optimal cutting temperature compound
DAPI: 4,6-diamidino-2-phenylindole
TdT: Terminal deoxynucleotidyl transferase
TUNEL: Terminal deoxynucleotidyl transferase (TdT) mediated dUTP-bitin nick end labeling
cDNA: Complementary DNA
A: Adenine deoxyribonucleotide
OD: Optical density
i.p.: Intraperitoneal
dpi: Days post-infection
BCA: Bicinchoninic acid
UMAP: Uniform Manifold Approximation and Projection
ExN: Excitatory neuron
InN: Inhibitory neuron
OEG: Olfactory ensheathing glial
OPCs: Oligodendrocyte precursor cells
Oligs: Oligodendrocytes
Astro: Astrocytes
Bergm: Bergmann glial cells
ABCs: Arachnoid barrier cells
VLMCs: Vascular leptomeningeal cells
Epen: Ependymal cells
CP: Choroid plexus cells
Endo: Endothelial cells
Peri: Pericytes
Micro: Microglia
Macro: Macrophages
Mono: Monocytes
Neut: Neutrophils
CX: Cerebral cortex
OB: Olfactory bulb
CA: Cornu Ammonis
DG: Dentate Gyrus
DEGs: Differentially expressed genes
KEGG: Kyoto Encyclopedia of Genes and Genomes
UMIs: Unique molecular identifiers
NS5: Non-structural protein 5
VGNAs: Viral genome nucleus accumulated cells
FasL, FASLG, CD95L: Fas ligand
TNF: Tumor necrosis factor
NK: Natural killer
MOI: Multiplicity of infection
hpi: Hours post-inoculation
Snap25: Synaptosomal-associated protein 25
OFT: Open field test
HPT: Hot plate test
MTase: N-terminal methyltransferase
RdRp: RNA-dependent RNA-polymerase
GEO: Gene Expression omnibus database
SRA: Sequence Read Archive database
