# Supplementary material for: Decoding dengue’s neurological assault: insights from single-cell CNS analysis in an immunocompromised mouse model
Source: J Neuroinflammation. 2025 Mar 4;22:62. doi: 10.1186/s12974-025-03383-w (PMC11877810; doi:10.1186/s12974-025-03383-w)
Supplement: Supplementary file 2 — Supplementary Material 2 [file 12974_2025_3383_MOESM2_ESM.pdf]

**Supplementary figure 1**

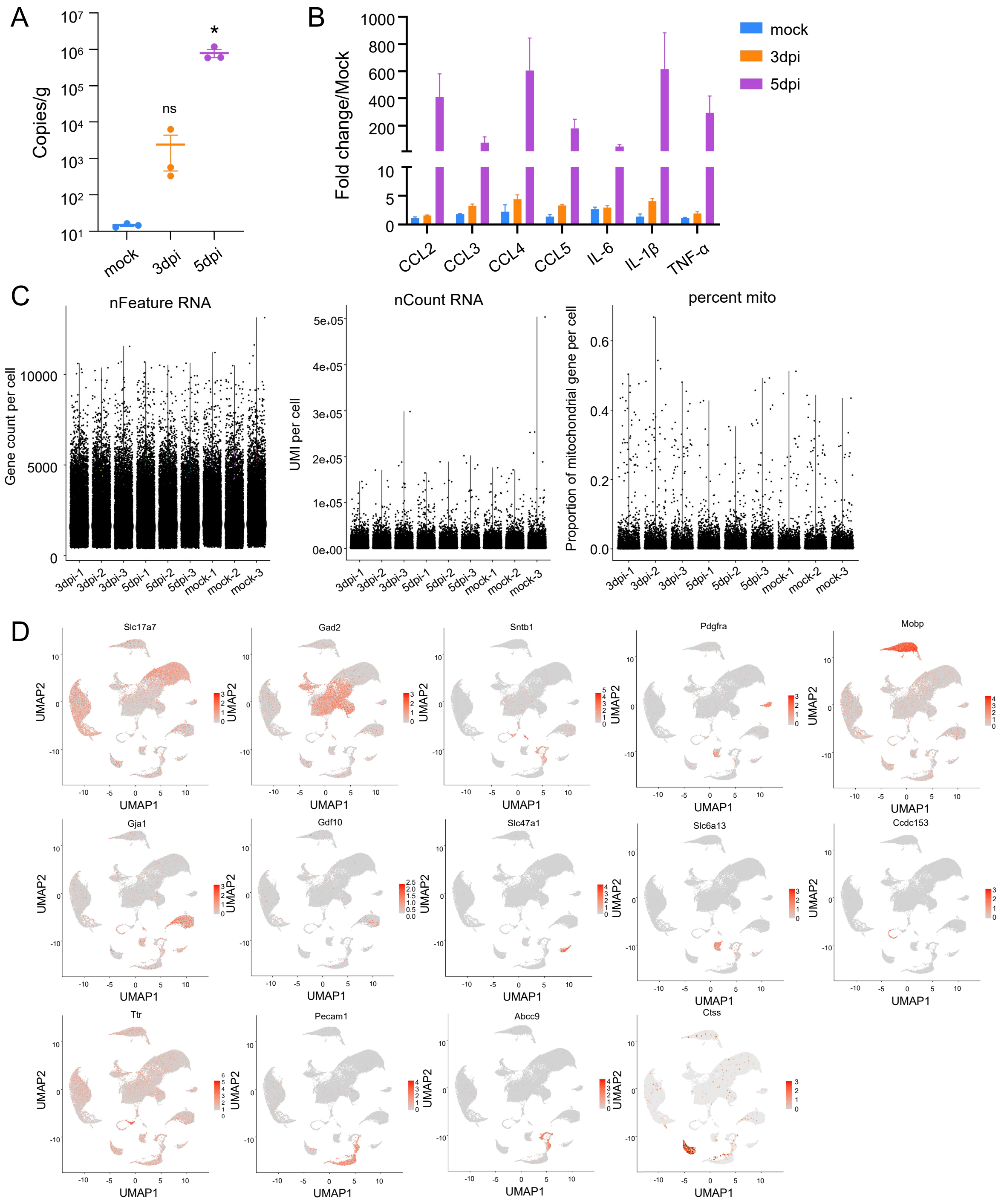


**SF 1. Quality of effectively infected mice brain samples for snRNA-seq.** (A) Virus loads in mice brain samples detected by qPCR. (B) Expression of inflammatory factors and chemokines in mice brain samples detected by qPCR (mock, n=3; 3dpi, n=3; 5dpi, n=3). (C) Number of genes detected in each cell (left). Total expression of all genes detected in each cell (medium). Proportion of detected mitochondrial genes (right). (D) Features of example markers of identified cell types. * indicates p value < 0.05, ** indicates p value < 0.01. Data are presented as mean ± sem.

**Supplementary figure 2**

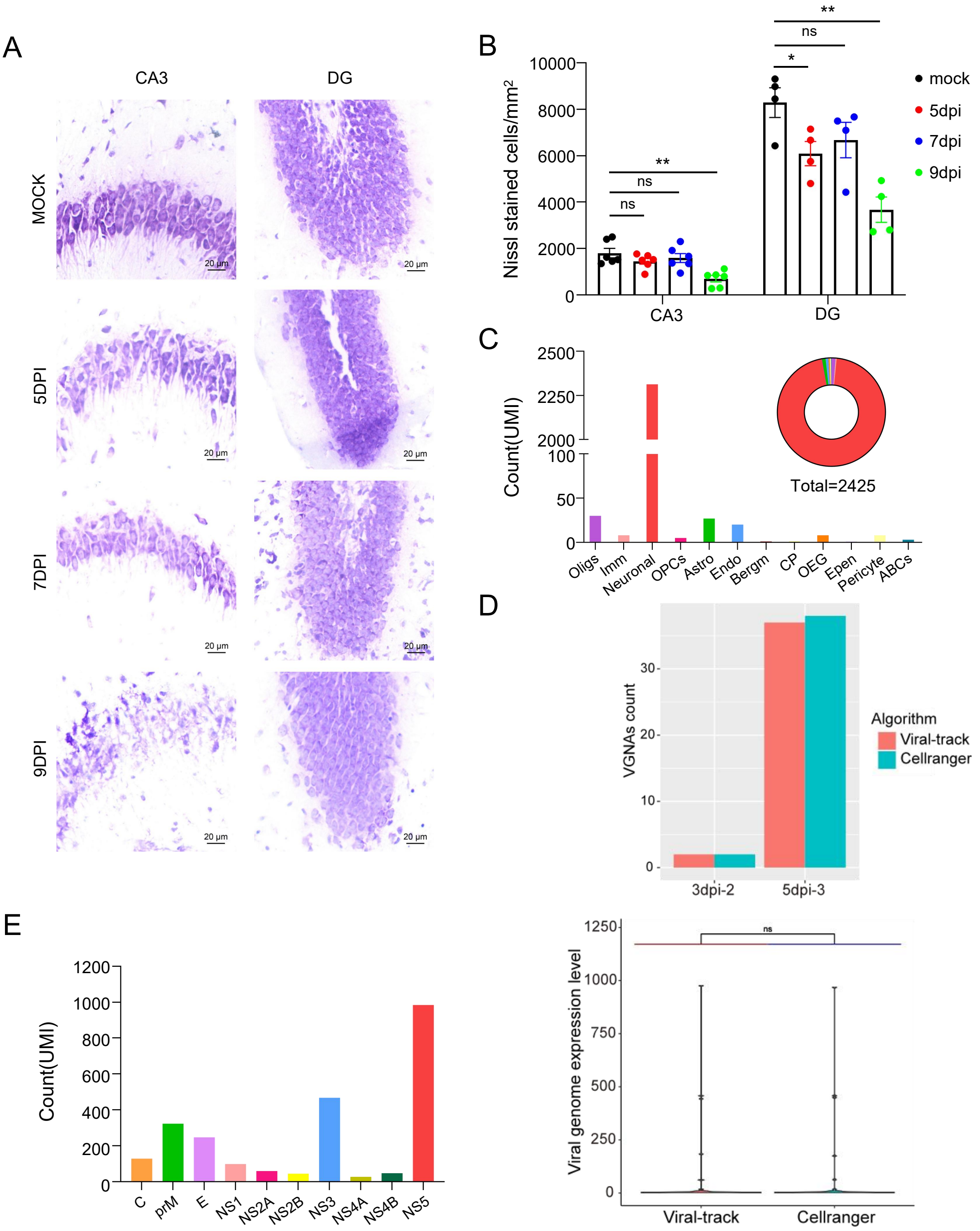


**SF 2. Characteristics of infected brain tissue and DENV.** (A) Representitive Nissl staining images of DENV infected mice brain tissue at HP area. Scale bar: 20 μm. (B) Statistical graphic of Nissl staining in (A). (C) Statistical graphic of the density of viral genome fragments in each cell type. (D) Statistical graphic of viral genome counts and expression level analyzed by viral-track and cellranger. (E) Counts of different regions of viral genome detected by snRNA-seq. * indicates p value < 0.05, ** indicates p value < 0.01. *** indicates p value < 0.001. Data are presented as mean ± sem.

## Supplementary figure 3

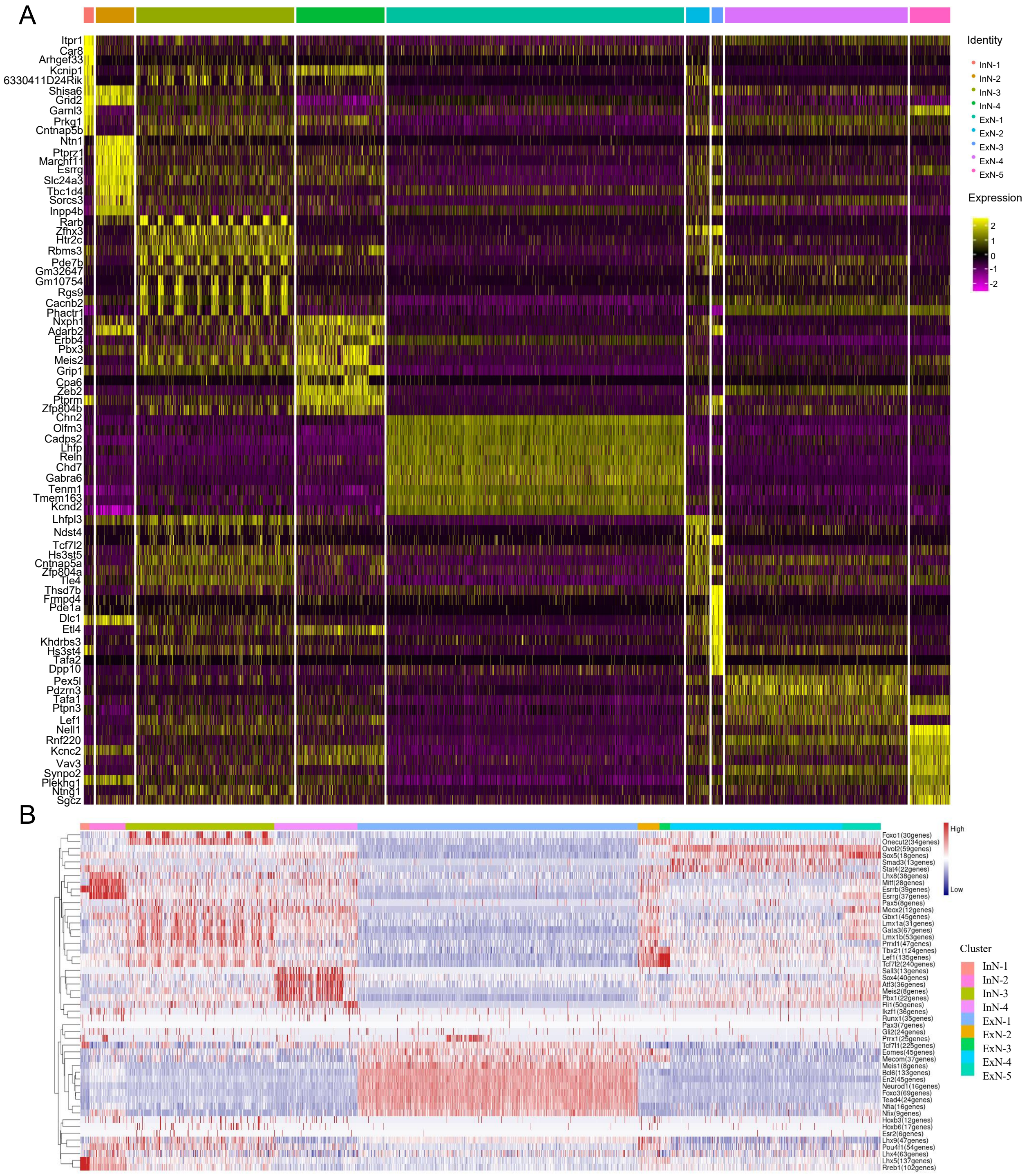


**SF 3. Characteristics of neuronal subclusters.** (A) Heat map of high expression characteristic genes in each subcluster. (B) Single-cell regulatory network inference and clustering (SCENIC) of each neuronal subcluster.

## Supplementary figure 4

A

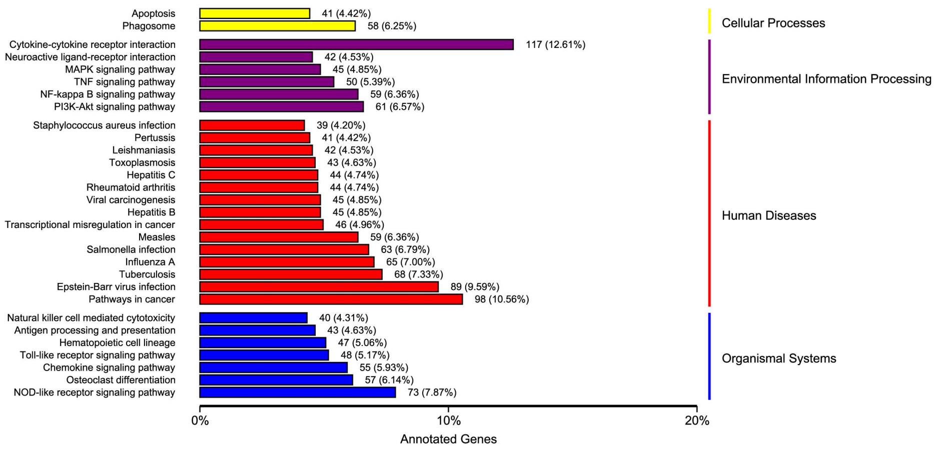


B

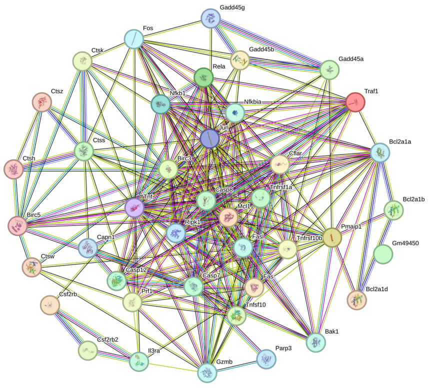


**Reactome Pathway**

| *pathway* | *description* | *Count in network* | *strength* | *False discovery fate* |
|---|---|---|---|---|
| MMU-75158 | TRAIL signaling | 4 of 5 | 2.63 | 1.24e-07 |
| MMU-75157 | FasL/ CD95L signaling | 3 of 4 | 2.6 | 9.64e-06 |
| MMU-69416 | Dimerization of procaspase-8 | 7 of 10 | 2.57 | 3.04e-13 |
| MMU-5218900 | CASP8 activity is inhibited | 7 of 10 | 2.57 | 3.04e-13 |

**Tissue expression**

| *tissue* | *description* | *Count in network* | *strength* | *False discovery fate* |
|---|---|---|---|---|
| BTO:0001170 | Resting cell | 2 of 2 | 2.73 | 0.0016 |
| BTO:0000289 | Cytotoxic T-lymphocyte | 3 of 15 | 2.03 | 0.00061 |
| BTO:0003292 | RAW-264.7 cell | 4 of 22 | 1.99 | 5.44e-05 |
| BTO:0000914 | Natural killer cell | 3 of 23 | 1.84 | 0.0016 |

C

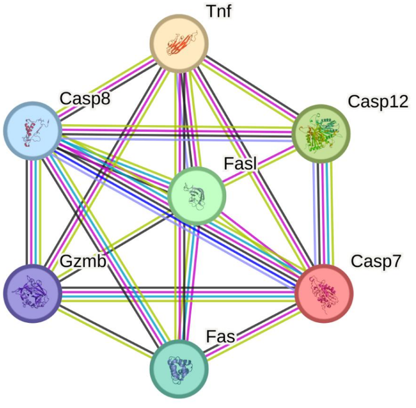


| Node | Node degree |
|---|---|
| Tnf | 28 |
| Casp8 | 26 |
| Fasl | 23 |
| Casp7 | 20 |
| Fas | 18 |
| Gzmb | 12 |
| Casp12 | 11 |

**SF 4. Transcriptome characteristics of olfactory bulb (OB) in DENV infected mice.** (A) Enriched KEGG pathways of DENV infected mice OB at 5dpi. (B) STRING interaction network analysis of genes enriched in apoptosis pathway from (A) (left) and top 4 of reactome pathway and tissue expression analysis (right). (C) Interaction diagram of core gene networks which enriched in apoptotic signaling pathway from DENV infected mouse OB RNA-seq detection.

**Supplementary figure 5**

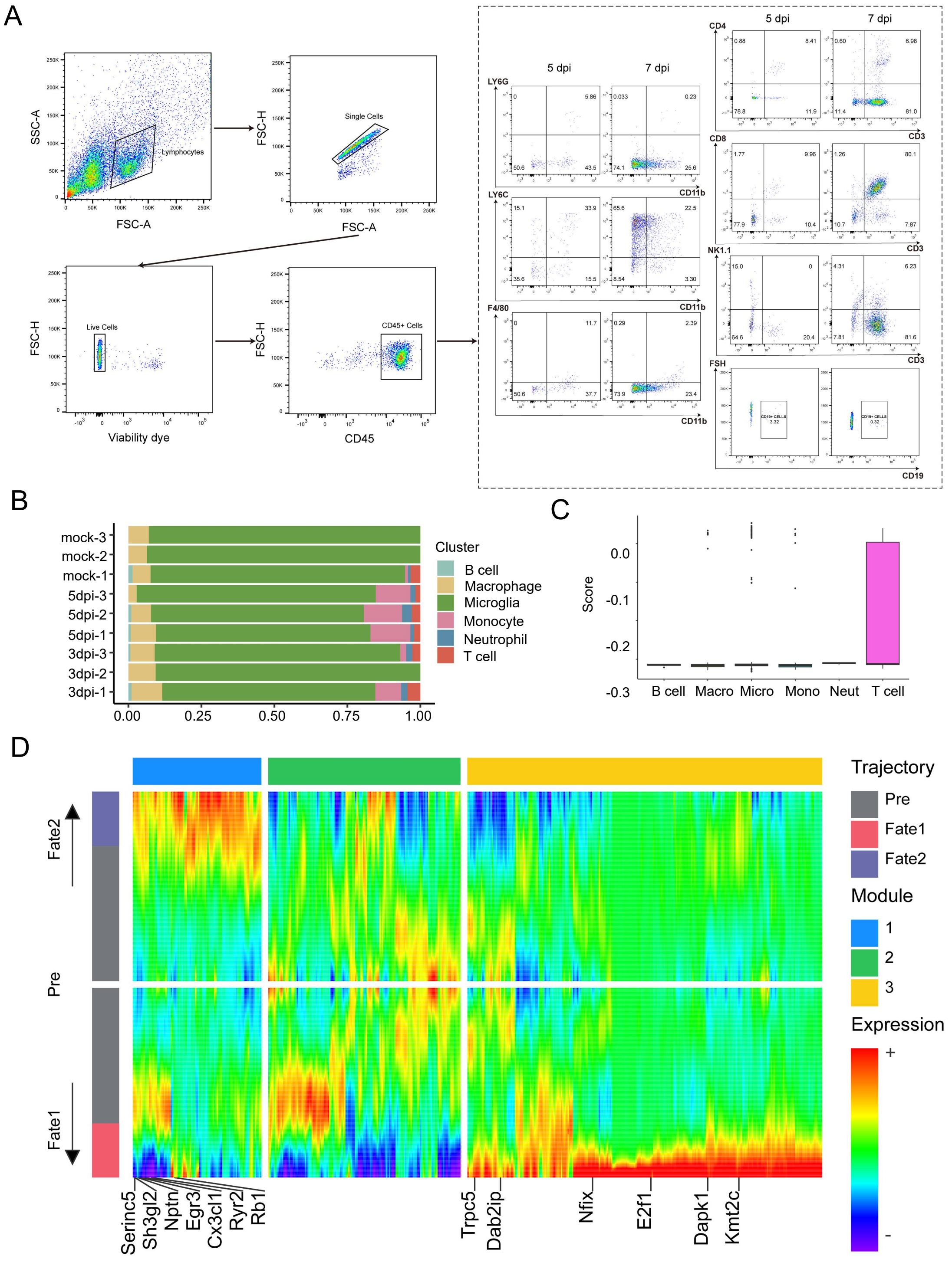


**SF 5. Characteristics of infiltrated immune cells in DENV infected mice brain tissue.** (A) Infiltrated immune cells in brain detected by flow cytometry detected at 5dpi and 7dpi. (B) The proportion of immune cells at 3dpi and 5dpi in each sample. (C) Cytokine production involved in inflammatory response of T cells which characterized in snRNA-seq study by single sample gene set enrichment analysis (ssGSEA). (D) The BEAM plot of pseudotime trajectory of the InN-4 cluster.

## Supplementary figure 6

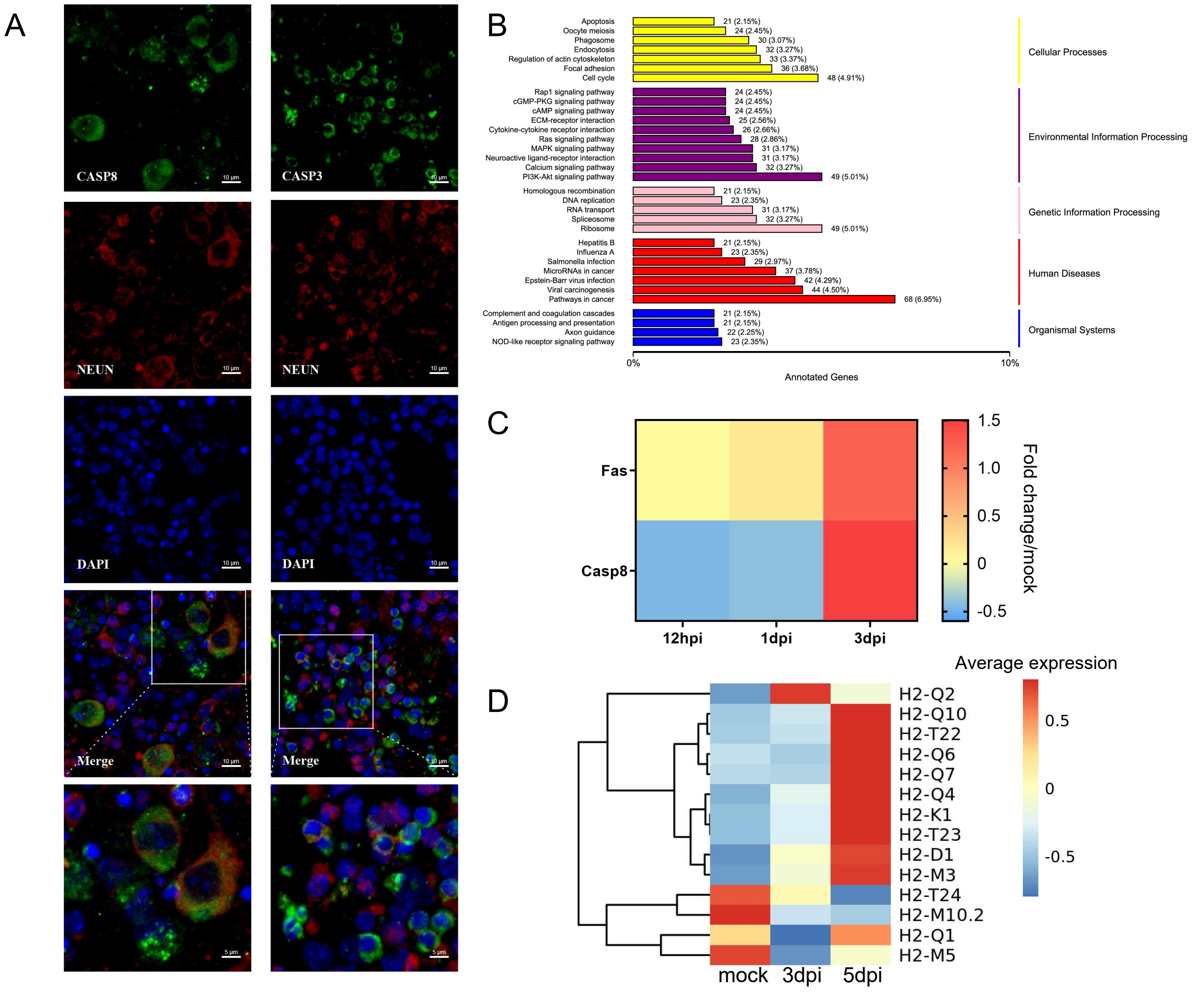


**SF 6. Characteristics of Fas-FasL axis mediated apoptosis in tissues and cell lines.** (A) Representative immunofluorescence images of downstream factors of Fas-FasL pathway in DENV infected mice brain. Scale bar: 10 μm. Magnified view scale bar: 5um. (B) Enriched KEGG pathways of DENV infected neuro-2a strain cells at 3dpi. (C) Heat map of Fas and Casp8 transcriptome expression of neuro-2a strain cells infected with DENV at 12hpi, 1dpi, and 3dpi. (D) Expression of CD8$^{+}$ T cells specific antigen presentation molecule (MHC-I) related genes in neuronal cluster of snRNA-seq study.

## Supplementary figure 7

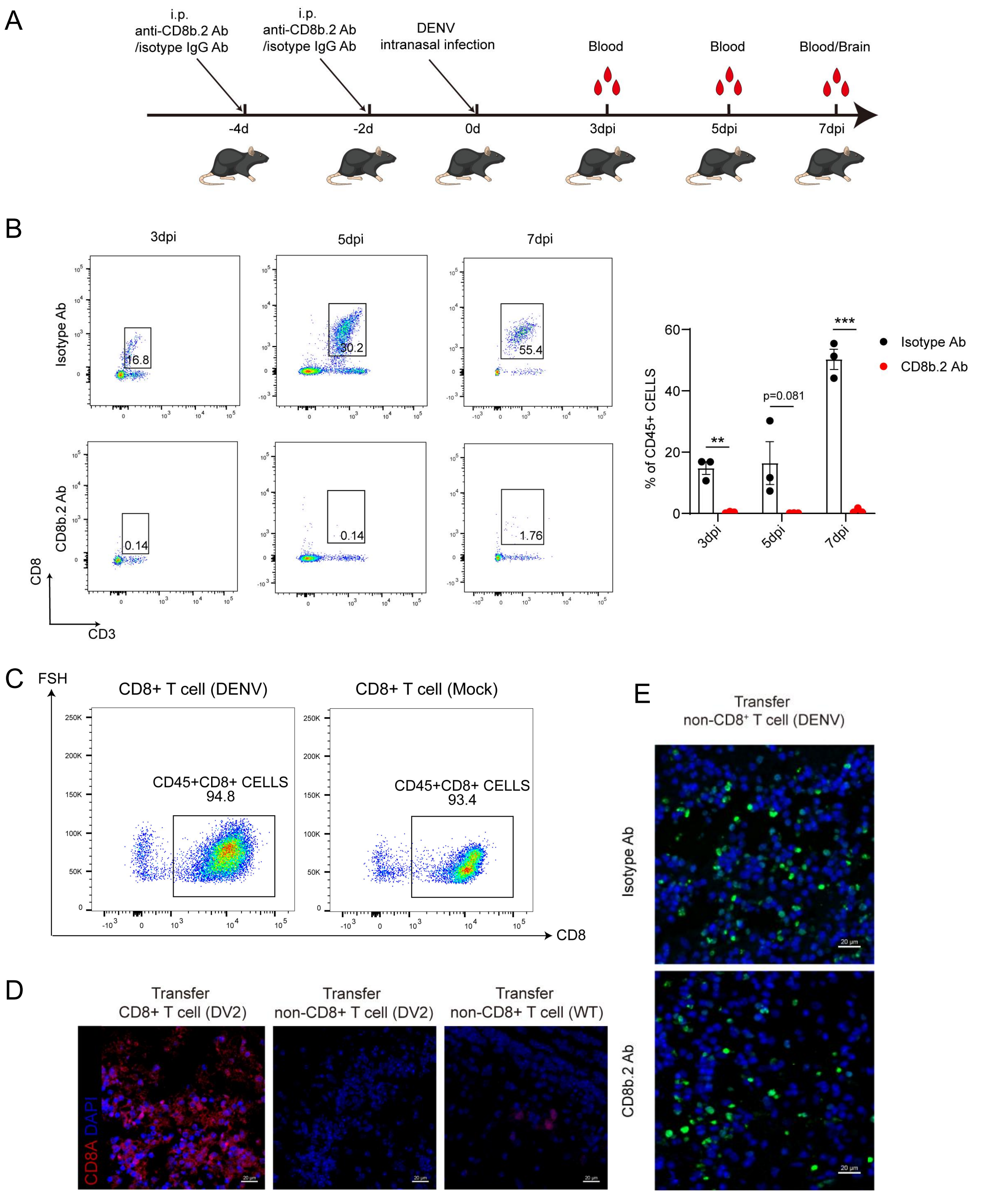


**SF 7. CD8⁺ T cell depletion and adoptive transfer.** (A) Schematic diagram of CD8⁺ T cell depletion and sampling after mouse DENV intranasal infection. (B) After intraperitoneal injection of CD8b.2 antibody or isotype antibody, CD3⁺CD8⁺ T cells in mouse blood were detected by flow cytometry at 3dpi, 5dpi and 7dpi. (C) Post-sort purity of CD8⁺ T cells analyzed by flow cytometry. (D) Representative immunofluorescence images of the CD8a in adoptive transferred mice OB at 7dpi. (E) Representative TUNEL staining images of OB in non-CD8⁺ T cell transferred mice at 7dpi. Scale bar: 20 μm. ** indicates p value < 0.01, *** indicates p value < 0.001. Data are presented as mean ± SEM.

## Supplementary figure 8

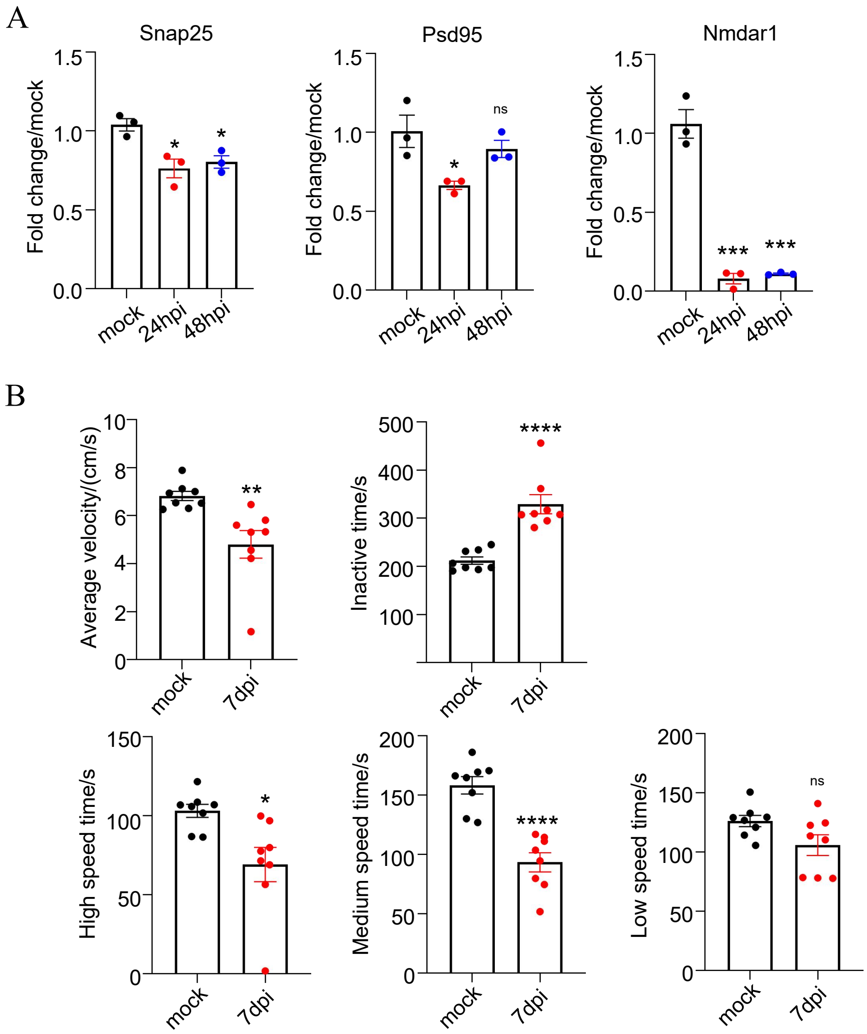


**SF 8. The effect of in vitro DENV infection on the expression of synaptic related genes and the characteristics of mice behavioral after infection.** (A) Synapse key structural organized genes mRNA expression level in neuro-2a after DENV infection. (B) OF behavioral profile of the mock-infected mice and DENV infected mice at 7dpi. Statistical graphic of average velocity, inactive time, high speed time, medium speed time and low speed time (form left to right). * indicates p value < 0.05, ** indicates p value < 0.01. *** indicates p value < 0.001. **** indicates p value < 0.0001. Data are presented as mean ± sem.
