# Supplementary material for: Decoding dengue’s neurological assault: insights from single-cell CNS analysis in an immunocompromised mouse model
Source: J Neuroinflammation. 2025 Mar 4;22:62. doi: 10.1186/s12974-025-03383-w (PMC11877810; doi:10.1186/s12974-025-03383-w)
Supplement: Supplementary file 3 — Supplementary Material 3 [file 12974_2025_3383_MOESM3_ESM.docx]

**4Supplementary table 1**

| **Primer** | **Sequence** |
| --- | --- |
| DV2 | forward 5’- GCAGAATGCCCCAACACAAA -3’ |
|  | reverse 5’- ACAAAATACATCCTGCCTTTCTCT -3’ |
| CCL2 | forward 5’- TTAAAAACCTGGATCGGAACCAA -3’ |
|  | reverse 5’- GCATTAGCTTCAGATTTACGGGT -3’ |
| CCL3 | forward 5’- CTCCCAGCCAGGTGTCATTTT -3’ |
|  | reverse 5’- CAGGCATTCAGTTCCAGGTCAG -3’ |
| CCL4 | forward 5’- GCTGCTTTGCCTACCTCTCC -3’ |
|  | reverse 5’- TCGAGTGACAAACACGACTGC -3’ |
| CCL5 | forward 5’- TTTGCCTACCTCTCCCTCG -3’ |
|  | reverse 5’- CGACTGCAAGATTGGAGCACT -3’ |
| TNF-a | forward 5’-ATGGCCTCCCTCTCATCAGT -3’ |
|  | reverse 5’-TTTGCTACGACGTGGGCTAC -3’ |
| IL-1b | forward 5’-TGCCACCTTTTGACAGTGATG -3’ |
|  | reverse 5’-TGATGTGCTGCTGCGAGATT -3’ |
| IL-6 | forward 5’-ACCAGTGACTGAAAGACGCA -3’ |
|  | reverse 5’-TGGGGGAGGATGTTTGGATG -3’ |
| Plp1 | forward 5’- CCAGAATGTATGGTGTTCTCCC -3’ |
|  | reverse 5’- GGCCCATGAGTTTAAGGACG -3’ |
| Qk | forward 5’- CTGGACGAAGAAATTAGCAGAGT -3’ |
|  | reverse 5’- ACTGCCATTTAACGTGTCATTGT -3’ |
| Dcx | forward 5’- TTTGGACATTTTGACGAACGAGA -3’ |
|  | reverse 5’- GTGGGCACTATGAGTGGGAC -3’ |
| Auts2 | forward 5’- GGAGGTCTCGATCACAGCG -3’ |
|  | reverse 5’- TTCGGCTGAGGTGGACTCT -3’ |
| Adcy8 | forward 5’- GGCTTCCTACACCTTGACTGT -3’ |
|  | reverse 5’- ATGACCCCTCGGTAGCTGTAT -3’ |
| Pde1c | forward 5’- TTGGTCAAGCAATTAGAACGAGG -3’ |
|  | reverse 5’- CAGCAGTCGCCTTGTTTCAT -3’ |
| Gad1 | forward 5’- AACGTATGATACTTGGTGTGGC -3’ |
|  | reverse 5’- CCAGGCTATTGGTCCTTTGTAAG -3’ |
| Fas | forward 5’- ATGCACACTCTGCGATGAAG-3’ |
|  | reverse 5’- CAGTGTTCACAGCCAGGAGA-3’ |
| Gzmb | forward 5’- TCTCGACCCTACATGGCCTTA -3’ |
|  | reverse 5’- TCCTGTTCTTTGATGTTGTGGG -3’ |
| Casp8 | forward 5’-ATGGCGGAACTGTGTGACTCG-3’ |
|  | reverse 5’-GTC ACCGTGGGATAGGATACAGCA-3’ |
| Casp12 | forward 5’-TAGGGGAAAGTGCGAGTTTCA-3’ |
|  | reverse 5’-GGGCCAATCCAGCATTTACCT-3’ |
| Becn1 | forward 5’- ATGGAGGGGTCTAAGGCGTC -3’ |
|  | reverse 5’- TGGGCTGTGGTAAGTAATGGA -3’ |
| Rb1cc1 | forward 5’- GACACTGAGCTAACTGTGCAA -3’ |
|  | reverse 5’- GCGCTGTAAGTACACACTCTTC -3’ |
| Atg101 | forward 5’- AGGAGGGCACGTACTCCATAG -3’ |
|  | reverse 5’- GCACATAGGTGAAGTCGATGAAG -3’ |
| C9orf72 | forward 5’- TTGGCGGCTACCTTTGCTTAC -3’ |
|  | reverse 5’- CATTCCAGTTTCCGTCGAAGA -3’ |
| Slc39a14 | forward 5’- GAGTGGGCCGGGATAATGTTT -3’ |
|  | reverse 5’- GAGATCGCTCGCTCAAGTTGT -3’ |
| Pcbp1 | forward 5’- GACGCCGGTGTGACTGAAA -3’ |
|  | reverse 5’- GTCAGCGTGATGATCCTCTCC -3’ |
| Mlkl | forward 5’- TTAGGCCAGCTCATCTATGAACA -3’ |
|  | reverse 5’- TGCACACGGTTTCCTAGACG -3’ |
| Ripk3 | forward 5’- CAGTGGGACTTCGTGTCCG -3’ |
|  | reverse 5’- CAAGCTGTGTAGGTAGCACATC -3’ |
| Gsdmd | forward 5’- ATGCCATCGGCCTTTGAGAAA -3’ |
|  | reverse 5’- AGGCTGTCCACCGGAATGA -3’ |
| Casp1 | forward 5’-ACAAGGCACGGGACCTATG-3’ |
|  | reverse 5’-TCCCAGTCAGTCCTGGAAATG-3’ |
| FasL | forward 5’-GCAGAAGGAACTGGCAGAAC-3’ |
|  | reverse 5’-TTAAATGGGCCACACTCCTC-3’ |
| Tnfsf10 | forward 5’- ATGGTGATTTGCATAGTGCTCC -3’ |
|  | reverse 5’- GCAAGCAGGGTCTGTTCAAGA -3’ |
| Snap25 | forward 5’- GGCTGACCAGCTGGCTGAT -3’ |
|  | reverse 5’- TGCCAGCATCTTTACTCTCTTCAA -3’ |
| Psd95 | forward 5’- AGTCTGTGCGAGAGGTAG -3’ |
|  | reverse 5’- GGATGAAGATGGCGATAGG -3’ |
| Nmdar1 | forward 5’- CGCGAGATCTCTGGGAAT -3’ |
|  | reverse 5’- GACTCGTTCTTGCCGTTGATTA -3’ |
| Bid | forward 5’- GCCGAGCACATCACAGACC |
|  | reverse 5’-TGGCAATGTTGTGGATGATTTCT |
| Bax | forward 5’-AGACAGGGGCCTTTTTGCTAC |
|  | reverse 5’- AATTCGCCGGAGACACTCG |
| Bak1 | forward 5’-CAGCTTGCTCTCATCGGAGAT |
|  | reverse 5’- GGTGAAGAGTTCGTAGGCATTC |
| Trp53 | forward 5’-CCCCTGTCATCTTTTGTCCCT |
|  | reverse 5’- AGCTGGCAGAATAGCTTATTGAG |
| Ephb1 | forward 5’-CCTCCTCCTATGGACTGCCC |
|  | reverse 5’- AAGGCCGTGAAGTCTGGGATA |
| Nr4a2 | forward 5’-GTGTTCAGGCGCAGTATGG |
|  | reverse 5’- TGTATTCTCCCGAAGAGTGGTAA |
| Ptch1 | forward 5’-GCCTTCGCTGTGGGATTAAAG |
|  | reverse 5’- CTTCTCCTATCTTCTGACGGGT |
| Dlx5 | forward 5’-GTCCCAAGCATCCGATCCG |
|  | reverse 5’- GCGATTCCTGAGACGGGTG |
| Fgf13 | forward 5’-TCGCTCATCCGGCAAAAGAG |
|  | reverse 5’- TGTCGGCTGTATAGTTTGGTAAC |
| Map2 | forward 5’-GCCAGCCTCAGAACAAACAG |
|  | reverse 5’- AAGGTCTTGGGAGGGAAGAAC |
| Aif1 | forward 5’-CTTGAAGCGAATGCTGGAGAA |
|  | reverse 5’- GGCAGCTCGGAGATAGCTTT |
| pcna | forward 5’-TTGCACGTATATGCCGAGACC |
|  | reverse 5’- GGTGAACAGGCTCATTCATCTCT |
| Mki67 | forward 5’-ATCATTGACCGCTCCTTTAGGT |
|  | reverse 5’- GCTCGCCTTGATGGTTCCT |
| Ubb | forward 5’- TTCGGTCTGCATTCCCAGTG -3’ |
|  | reverse 5’- AACTTAAATTGGGGCAAGTGGC -3’ |
